# Structured Personalized Oxygen and Supportive Therapies for Dyspnea in Oncology (SPOT-ON): A personalized randomized clinical trial protocol

**DOI:** 10.1371/journal.pone.0336691

**Published:** 2025-12-02

**Authors:** David Hui, Min Ji Kim, Saji Thomas, Gautam Sachdev, Sanjay Shete, Nisha Rathi, Nicole Hensch, Vera De La Cruz, Amy Ontai, Donald A. Mahler, Jie Li, Eduardo Bruera

**Affiliations:** 1 Department of Palliative, Rehabilitation and Integrative Medicine, The University of Texas MD Anderson Cancer Center, Houston, Texas, United States of America; 2 Center for Goal Concordant Care Research, The University of Texas MD Anderson Cancer Center, Houston, TX, USA; 3 Department of General Oncology, The University of Texas MD Anderson Cancer Center, Houston, TX, USA; 4 Department of Critical Care Medicine, The University of Texas MD Anderson Cancer Center, Houston, Texas, United States of America; 5 Department of Biostatistics, The University of Texas MD Anderson Cancer Center, Houston, Texas, United States of America; 6 Department of Medicine, Geisel School of Medicine at Dartmouth, Hanover, New Hampshire, United States of America; 7 Department of Cardiopulmonary Sciences, Rush University, Chicago, Illinois, United States of America; PLOS: Public Library of Science, UNITED KINGDOM OF GREAT BRITAIN AND NORTHERN IRELAND

## Abstract

**Background:**

Low-flow supplemental oxygen (LFSO), high-flow nasal cannula (HFNC), and non-invasive ventilation (NIV) are often used for palliation of dyspnea in hospitalized patients. However, it is unclear how patients prefer to use these modalities and how they can be personalized for optimal dyspnea relief. The objective of this randomized clinical trial is to compare the effect of a personalized respiratory therapist (RT)-led intervention, termed SPOT-ON, and enhanced usual care on dyspnea intensity in both hypoxemic and non-hypoxemic hospitalized patients with cancer.

**Methods:**

In this two-arm, parallel group, partially blinded, waitlist-controlled randomized clinical trial, hospitalized patients with advanced cancer and at least moderate dyspnea intensity at rest (numeric rating scale [NRS] ≥4/10) will be randomized 1:1 to receive SPOT-ON immediately or after a 72-hour waiting period. The SPOT-ON intervention consists of a time-limited trial of LFSO, HFNC, and NIV for 10 minutes each, followed by personalized deployment of these modalities to tailor device settings and timing and duration of use based on patient preference over 72 hours. The waitlist control group will receive structured dyspnea education after enrollment and usual care. The primary outcome will be change in dyspnea NRS intensity from baseline to 24 hours. Secondary outcomes include dyspnea unpleasantness, vital signs, symptom burden, health-related quality of life, adverse events, pattern of device use, and hospital outcomes. We calculated that 150 patients (75 hypoxemic and 75 non-hypoxemic) will provide 80% power to detect a 1.2-point difference between groups with α = 0.025, assuming a standard deviation of 1.5 and 20% attrition.

**Discussion:**

Successful completion of this clinical trial could inform the use of oxygen and supportive modalities to reduce dyspnea. This novel personalized clinical trial methodology involving time-limited trials to tailor therapy based on patient preferences may inform the design of future supportive care clinical trials.

**Trial registration:**

Clinicaltrials.gov NCT06336642

## Introduction

Dyspnea, the sensation of difficulty breathing, is a highly distressing symptom in patients with cancer that is associated with impaired function, decreased quality of life, and shortened survival [1–4]. Affecting up to 70% of patients with cancer [5], dyspnea is highly prevalent, particularly in patients with intrathoracic malignancies and advanced disease [6,7]. Despite its high prevalence and negative impact, no therapies have been approved by the U.S. Food and Drug Administration (FDA) for dyspnea.

Several oxygen-based modalities have been found to reduce dyspnea in hospitalized patients, with low-flow supplemental oxygen (LFSO) being commonly offered. High-flow nasal cannula (HFNC, providing heated, humidified oxygen up to 100 L/min) and non-invasive ventilation (NIV, providing two levels of pressure on inspiration and expiration) represent two promising therapeutic options for dyspnea beyond traditional LFSO. In addition to oxygenation, both modalities can improve gas exchange, augment airway pressure, and reduce the work of breathing [3,4,8–11]. Several studies have reported that HFNC and NIV are effective in palliating dyspnea in patients with hypoxemia [9–13]**.** Preliminary clinical studies also support that HFNC may reduce dyspnea in patients without hypoxemia [14,15]. However, the lack of definitive studies to support the use of HFNC and NIV in patients with mild or no hypoxemia [16] coupled with uncertainties on how to personalize the use of these interventions for dyspnea means that HFNC and NIV are currently not offered to patients without severe hypoxemia, even when they have dyspnea.

To address this knowledge gap, we designed the Structured Personalized Oxygen and supportive Therapies for dyspnea in Oncology (SPOT-ON) intervention. This personalized, respiratory therapist (RT)-led intervention takes advantage of two fundamental insights. First, dyspnea is an acute symptom that responds rapidly to interventions; thus, short, timed therapeutic trials would allow clinicians to tailor treatments in real time and to personalize dyspnea interventions for each patient. Second, not all patients may tolerate or report a benefit from HFNC and NIV; consequently, it is important to personalize dyspnea treatment options to maximize therapeutic effect and adherence. We therefore hypothesize that the SPOT-ON intervention, incorporating time-limited trials of oxygen and supportive therapies, would be effective in reducing dyspnea in acutely ill, hospitalized patients with cancer. The objective of this National Cancer Institute-funded randomized clinical trial is to compare the effect of the personalized RT-led SPOT-ON intervention and enhanced usual care on dyspnea intensity in both hypoxemic and non-hypoxemic hospitalized patients with cancer.

## Methods

### Study design

**Fig 1:**
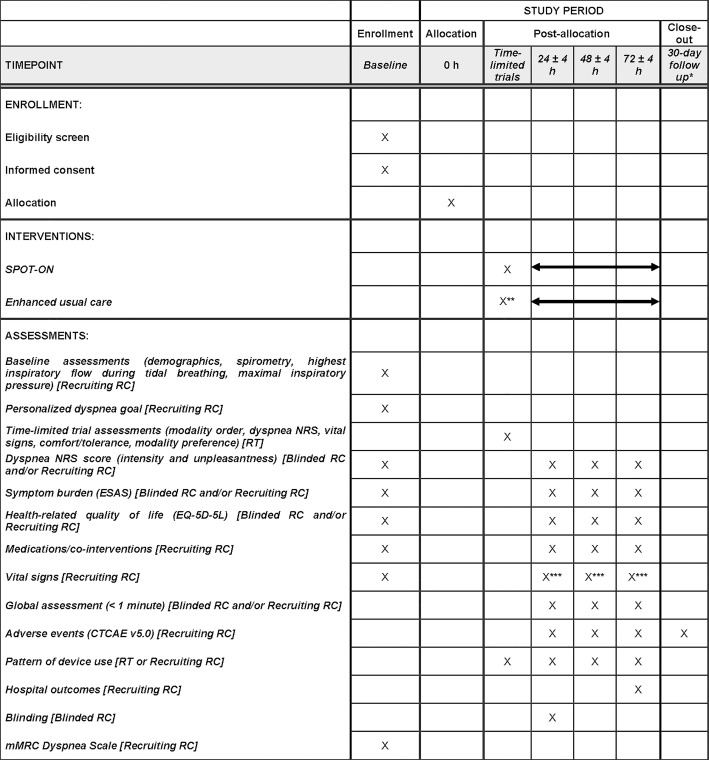
Schedule of enrollment, interventions, and assessments. Abbreviations: CTCAE, Common Terminology Criteria for Adverse Events; ESAS, Edmonton Symptom Assessment System; EQ-5D-5L, EuroQol-5 Dimension-5 Level; mMRC, Modified Medical Research Council; NRS, numeric rating scale; RC, research coordinator; RT, respiratory therapist; SPOT-ON, Structured Personalized Oxygen and Supportive Therapies for Dyspnea in Oncology.*Patients will be followed for 30 days after end of treatment or until death, whichever occurs first.**Patients will start SPOT-ON after 72 hours of enhanced usual care if they still meet eligibility criteria at that time. ***Vital signs will be measured every 8 hours during the trial.

**Fig 2:**
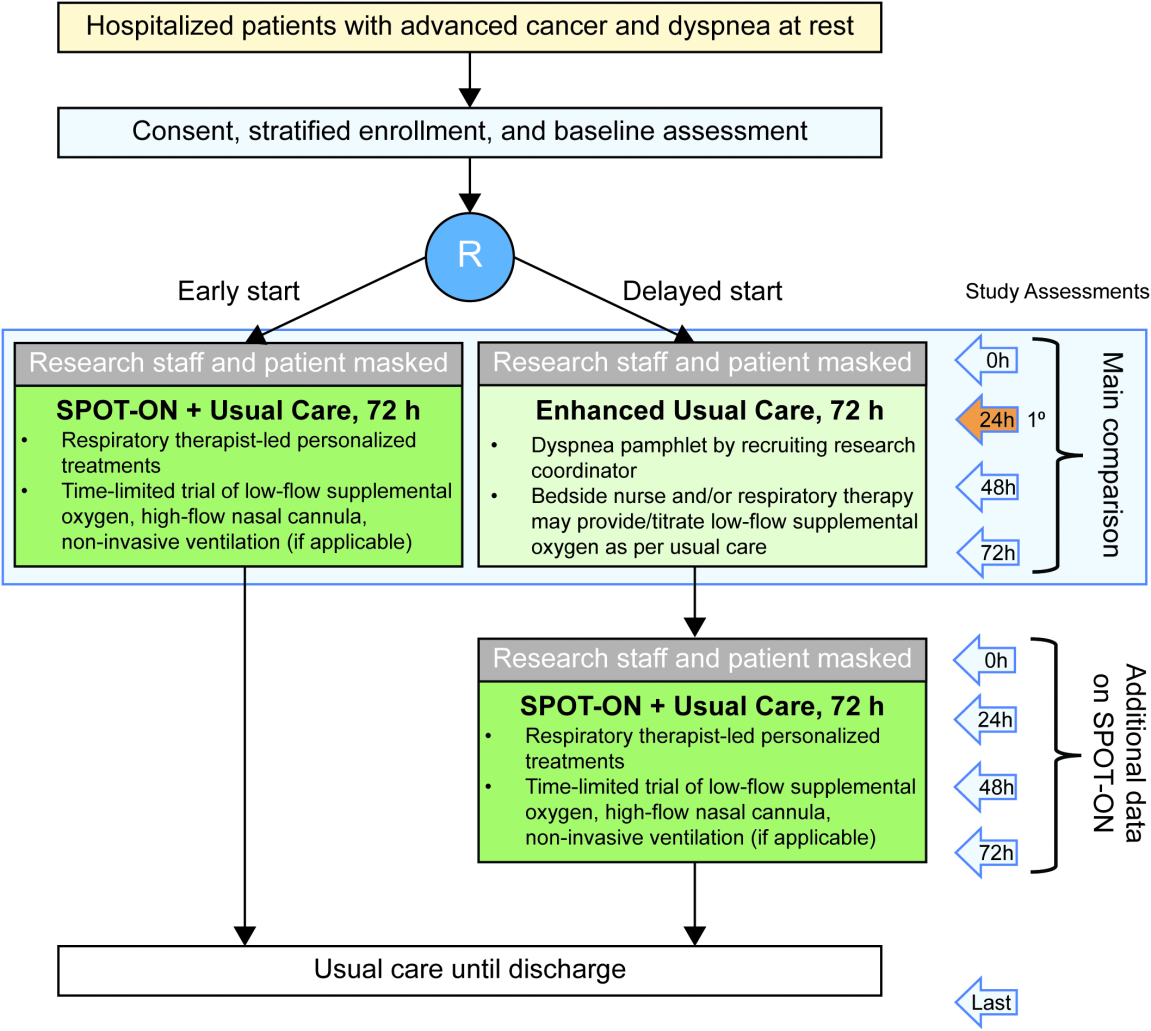
Study flow diagram. Abbreviation: SPOT-ON, Structured Personalized Oxygen and Supportive Therapies for Dyspnea in Oncology.

This is a two-arm, parallel group, partially blinded, waitlist-controlled randomized clinical trial examining the efficacy of the SPOT-ON intervention for dyspnea in cancer patients. The two arms of this study are the SPOT-ON intervention and enhanced usual care with waitlist control (Figs 1 and 2). The active intervention group will receive the SPOT-ON intervention immediately upon enrollment for 72 hours. The waitlist control group will receive usual care for 72 hours along with educational material on dyspnea as an attention control; they will start the SPOT-ON intervention after the 72-hour waiting period if they still meet eligibility requirements at that time.

### Eligibility criteria

Inclusion criteria include diagnosis of advanced cancer (metastatic, locally advanced, recurrent, or incurable); age 18 or older; admission to a medical floor; dyspnea intensity at rest of at least 4/10 on an 11-point numeric rating scale (NRS), where 0 = none and 10 = worst [17]; and ability to speak English or Spanish.

Exclusion criteria include hemodynamic instability requiring active Medical Emergency Rapid Intervention Team or Intensive Care Unit (ICU) team involvement; delirium as per clinical team’s assessment in the Electronic Health Record (EHR); severe hypoxemia (saturation of peripheral oxygen [SpO_2_] < 90% despite supplemental oxygen of up to 6 L/min); continuous positive airway pressure (CPAP) use for obstructive sleep apnea, actively using >10 hours a day; respiratory failure necessitating mechanical ventilation, HFNC, or NIV; planned thoracentesis within 72 hours of enrollment; and known pregnancy.

These eligibility criteria are intentionally broad with few exclusion criteria because patients with contraindications to a particular intervention will simply skip that treatment option as in routine clinical practice, which will help to maximize recruitment and generalizability.

### Recruitment and setting

Patients admitted to The University of Texas MD Anderson (MD Anderson) will be screened systematically on the above eligibility criteria based on a combination of EHRs and in-person assessments. If patients are eligible and interested, we will ask them to sign an informed consent document after obtaining permission from the attending team during the same hospital visit. Participants will receive a $50 gift card upon completion of the study to compensate them for their time and effort.

### Randomization and blinding

Allocation to the study groups will be determined at random in a 1:1 ratio using the Clinical Trial Conduct website developed by the MD Anderson Department of Biostatistics. Randomization will be stratified by baseline dyspnea intensity at rest (≤ 6/10 vs. ≥ 7/10) in both the hypoxemic and non-hypoxemic cohorts.

Research staff conducting the outcome assessments during the first 72 hours will be blinded to treatment assignment (Table 1). While patients will know when they are participating in the SPOT-ON intervention, we will state in the consent document that we will randomly allocate a time to start SPOT-ON, but we will not disclose the specific timing of the two study groups (i.e., immediately vs. 72 hours later). This strategy, in conjunction with the educational material, will allow us to partially blind the patients to their treatment group assignment. It will also provide all patients with the opportunity to try the SPOT-ON intervention.

**Table 1:** Blinding procedures in SPOT-ON randomized clinical trial.

Individuals	Blinding status	Roles and responsibilities
Recruiting research coordinator (not blinded)	Unblinded but will not be conducting outcome assessments	Obtain assignment from randomization website; conduct baseline assessments only; notify blinded research coordinators and research respiratory therapists to start their assigned tasks; provide education for the enhanced usual care group. For patients randomized to enhanced usual care, all assessments conducted by the blinded coordinator during the enhanced usual care phase will subsequently be conducted by the recruiting coordinator when they receive SPOT-ON 72 hours later.
Blinded research coordinator (blinded)	Unaware if patient is on wait-list control or not; masked to timing of enrollment	Conduct all key study assessments for three days, using telephone to maintain blinding (SPOT-ON or usual care, intervention group or wait-list control)
Research respiratory therapist (blinded)	Unaware if patient is on wait-list control or not; masked to timing of enrollment	Conduct focused respiratory therapy and assessments during SPOT-ON intervention
Patient (partially blinded)	Will not be aware of the two different study groups in the study (difference in start time)	Work with research respiratory therapists to complete intervention and research coordinators to complete assessments
Principal investigator (not blinded)	Unblinded but will not be involved in intervention delivery nor outcome assessments	Overall training and monitoring of study Monitor SPOT-ON fidelity

Abbreviation: SPOT-ON, Structured Personalized Oxygen and Supportive Therapies for Dyspnea in Oncology.

### SPOT-ON intervention

**Fig 3:**
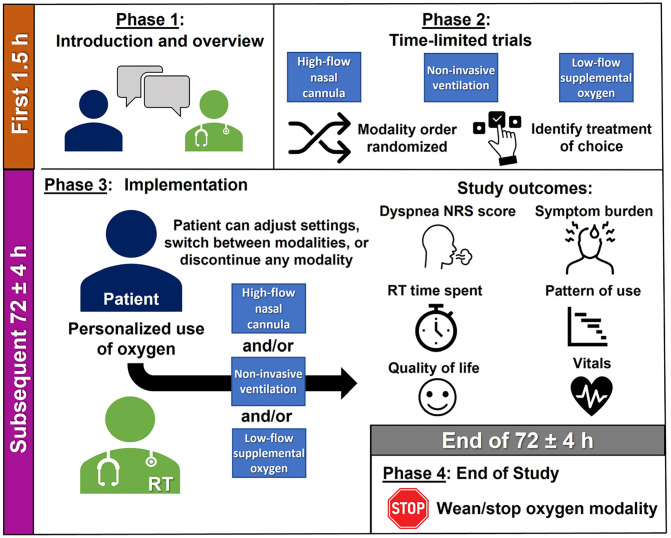
SPOT-ON study logistics. Abbreviations: NRS, Numeric Rating Scale; RT, Respiratory Therapist. Respiratory therapists will identify preferred dyspnea treatment choices with individual patients based on time-limited trials and offer personalized use of oxygen using the specified modalities over the remaining 72 ± 4 hours.

The SPOT-ON intervention consists of four phases conducted across 72 hours (Fig 3).

The first phase (Phase 1) consists of an orientation led by a blinded RT. The orientation will begin with an introduction to the patient and caregiver(s). The RT will review the patient’s prior experience with oxygen and supportive therapies and verify any contraindications. Specifically, HFNC and NIV will not be offered to patients with any of the following criteria: fixed obstruction of upper airway (e.g., tumor); facial, upper airway, or upper gastrointestinal surgery within the past week; inability to protect the airway; or copious respiratory secretions. NIV will not be offered to patients with gastrointestinal bleed; bowel obstruction; or pneumothorax without intercostal drain insertion. An overview of the study intervention, blinding procedure, and study expectations will be discussed, as well as the patient’s respiratory care goals.

Phase 2 involves time-limited trials of the three available dyspnea treatment modalities: LFSO, HFNC, and NIV. Patients will be asked to select one of six unlabeled envelopes, with each envelope specifying a unique order in which time-limited trials of LFSO, HFNC, and NIV will be delivered. Each modality will be tested for up to 10 minutes, with the goal of identifying the lowest possible setting to optimize dyspnea relief and overall comfort after optimization by the RT. Additionally, the RT will assess dyspnea, modality tolerance, vitals, and comfort in each time-limited trial. After all modalities have been tested, patient modality preference and treatment(s) of choice will be determined with the RT.

The third phase (Phase 3) implements the patient’s dyspnea treatment preferences for a total intervention period of 72 hours, with time 0 set as immediately before the start of Phase 1. The RT will visit the patient twice daily to provide personalized use of oxygen and supportive modalities. During these visits, patients may choose to adjust settings, switch between modalities, or discontinue any modality. Additional visits may be requested based on patient needs (up to 3 times/day). During the study intervention, the RT will document the pattern of modality use over the 72 hours, time spent with the patient, and vitals.

The final phase (Phase 4) of the SPOT-ON approach involves weaning and/or stopping the patient’s oxygen modality. The patient may discuss further treatment options with their primary team.

A research RT with dedicated SPOT-ON intervention training will be available from 8 am – 5 pm 7 days a week to deliver Phase 1 and 2 in a standardized manner. The research RT will also deliver Phase 3 and 4 except for after hours, when other clinical RTs will follow the treatment plan outlined by the research RT. The study will continue until end of study intervention (72 hours), discharge, patient deterioration (hemodynamic instability, delirium, respiratory failure requiring HFNC or NIV, or mechanical ventilation), or withdrawal.

#### Treatment administration.

HFNC and NIV will be delivered using an FDA-approved Hamilton C1 ventilator with commercially available accessories, including nasal masks, facial masks, and nasal cannula (Hamilton Medical, Reno, Nevada). HFNC and NIV have been FDA-approved for the delivery of gases in clinical settings. Device settings are described in detail in Table 2.

**Table 2:** SPOT-ON device settings.

Modality	Initial setting	Titration
Low-flow supplemental oxygen	2 L/min or lowest rate to keep SpO_2_ ≥ 90%	Respiratory therapist will titrate FiO_2_ (room air or oxygen) and flow rate (2–6 L/min) based on patient comfort and effect on dyspnea intensity.
High-flow nasal cannula	FiO_2_ 50% (air if non-hypoxemic), 20 L/min, temperature 37°C	Respiratory therapist will titrate FiO_2_ (21–100%), flow rate (10–60 L/min), and temperature (34–37°C) based on patient comfort and effect on dyspnea intensity.
Non-invasive ventilation	FiO_2_ 50% (air if non-hypoxemic), inspiratory pressure 10 cmH_2_O, expiratory pressure 5 cmH_2_O, nasal mask	Respiratory therapist will titrate FiO_2_ (21–100%), inspiratory pressure 8–12 cmH_2_O, expiratory pressure 4–8 cmH_2_O, and use of nasal/facial mask based on patient comfort and effect on dyspnea intensity.

Abbreviations: cmH_2_O, centimeters of water; FiO_2_, fraction of inspired oxygen; SpO_2_, saturation of peripheral oxygen.

### Enhanced usual care

Patients randomized into the enhanced usual care arm will receive a 20-minute educational session by the recruiting research coordinator (RC) using a patient educational pamphlet on dyspnea. Over the next 72 hours, they will receive standard of care with treatments based on the attending teams’ recommendations, including but not limited to treatment of underlying causes, supportive care consultation, and other palliative measures.

After 72 hours of enhanced usual care, patients in this group will start the aforementioned SPOT-ON intervention, and the research RT will be asked to start treatment if patients still meet study eligibility criteria (e.g., dyspnea intensity at rest of at least 4 on a 0–10-point NRS). The control group is designed such that it reflects the current standard of care; enhanced usual care serves as an attention control, and the waitlist design provides patients with the opportunity to still try SPOT-ON.

### Assessments

Details of the study measures and timing of assessments are shown in Fig 1. Data collected at baseline will include patient demographics, cardiopulmonary comorbidities, Karnofsky performance status, medication use, spirometry, maximal inspiratory pressure, highest inspiratory flow during tidal breathing, vital signs, and evaluations using the Modified Medical Research Council (mMRC) Dyspnea Scale.

During the time-limited trials, vital signs (e.g., heart rate, respiratory rate, and blood pressure) and dyspnea will be assessed before and after each modality, patient comfort/tolerance and global assessment will be assessed after each modality, and patient modality preference will be assessed after time-limited trials have concluded (Fig 1). Patients will be asked to (1) rank the modalities they tested based on their overall comfort and tolerance, (2) indicate the modalities they would prefer to use for different instances of dyspnea (i.e., acute attacks vs. chronic), (3) specify if they would prefer not to use a particular modality, and (4) describe how they would like to use each of the devices over the next three days for maximum comfort.

Dyspnea intensity and unpleasantness will be assessed at baseline and at 24, 48, and 72 hours with a dyspnea NRS scale ranging from 0 to 10 [17] (Fig 1). The Minimal Clinically Important Difference (MCID) for dyspnea intensity and unpleasantness is a 1-point decrease for improvement on the NRS [18].

Global impression of change will be assessed to directly compare the level of dyspnea before and after the study intervention (Fig 1). Patients will be asked if they felt their dyspnea is better (a little better, somewhat better, moderately better, a good deal better, a great deal better, very great deal better), about the same, or worse (a little worse, somewhat worse, moderately worse, a good deal worse, a great deal worse, very great deal worse) [19–21].

Patients’ personalized dyspnea goal (PDG) will be established at baseline, prior to randomization (Fig 1). At enrollment, PDG will be assessed by asking the patient, “At what level of intensity would you feel comfortable, on a scale of 0 to 10, where 0 = no shortness of breath and 10 = worst possible?” Personalized dyspnea response is defined as dyspnea intensity NRS ≤ PDG [22,23].

Health-related quality of life will be assessed by the EuroQol-5 Dimension-5 Level (EQ-5D-5L) questionnaire, which is composed of the EQ visual analogue scale and a descriptive system comprised of five dimensions: mobility, self-care, usual activities, pain/discomfort, and anxiety/depression [24] (Fig 1). Patients rate each of these dimensions using five levels: no problems, slight problems, moderate problems, severe problems, and extreme problems [24]. EQ-5D-5L has been validated in English and Spanish [25,26].

Symptom burden will be assessed with the Edmonton Symptom Assessment System (ESAS) questionnaire, which measures 10 common symptoms in the past 24 hours (pain, fatigue, nausea, depression, anxiety, drowsiness, shortness of breath, appetite, sleep, and feeling of well-being) using a NRS ranging from 0–10, where 0 = none and 10 = worst possible [17,27]. The ESAS has been validated in English and Spanish [17,28] (Fig 1).

Pattern of device use will be recorded at the end of Phase 2 and daily during Phase 3 (Fig 1). This will additionally be recorded during the enhanced usual care phase. Device start and end times, in addition to device settings, will be noted.

Medications and co-interventions outside of the scope of the SPOT-ON intervention will be documented at baseline (immediately before Phase 1) and daily during Phase 3.

Vital signs (i.e., heart rate, respiratory rate, and blood pressure) will be measured during the intervention stage, at baseline (immediately before Phase 1), and every 8 hours in both the SPOT-ON and enhanced usual care waitlist group. We will import additional vital sign data from Epic during the hospital stay if already collected, as per routine clinical practice.

Hospital outcomes, including length of hospital stay, length of ICU admission, inpatient palliative care consultation, hospital mortality, and discharge location, will be recorded after discharge or end of study, whichever occurs first.

Adverse events will be documented using the Common Terminology Criteria for Adverse Events (CTCAE) v5.0 assessment in both the SPOT-ON and enhanced usual care interventions at 24 ± 4, 48 ± 4, and 72 ± 4 hours (Fig 1). Patients will also be followed for 30 days after end of treatment or until death, whichever occurs first. Patients removed from study for unacceptable adverse event(s) will be followed until resolution or stabilization of the adverse event.

Blinding will be assessed in patients and research staff conducting the outcome assessments at 24 ± 4 hours.

### Outcomes

The primary outcome is change in dyspnea intensity (NRS) between baseline and 24 hours in hypoxemic and non-hypoxemic hospitalized patients with cancer.

Secondary outcomes include change in dyspnea intensity (NRS), dyspnea unpleasantness (NRS), dyspnea response, vital signs, symptom burden, and health-related quality of life (EQ-5D-5L) over 72 hours; adverse events; patterns of device use; and hospital outcomes.

### Fidelity

All research staff, including the research RT and study coordinators, will undergo an 8-hour orientation before study activation to discuss (1) dyspnea, (2) the menu of interventions, (3) communication skills, (4) study procedures including blinding (Table 1), and (5) pitfalls and solutions. They will be provided with a research manual that includes standard operating procedures. Research staff will attend weekly Principal Investigator (PI) meetings. All clinical RTs (other than research RTs) will receive a 30-minute orientation, so they are aware of the study intervention and procedures. The PI or designate will assess SPOT-ON intervention fidelity with direct observation using a standardized checklist (first 3 consecutive patients, then randomly every 10 patients).

### Ethical considerations

This manuscript conforms to the Standard Protocol Items: Recommendations for Interventional Trials (SPIRIT) Checklist for a clinical trial protocol (S1 File). The protocol (S2 File), informed consent forms (S3 File), recruitment materials, and all participant materials were submitted to The University of Texas MD Anderson Cancer Center IRB for review. Approval of both the protocol and the consent forms were obtained before any participant was consented. Any amendments to the protocol will require review and approval by the IRB before the changes are implemented to the study. All changes to the consent forms will be approved by the IRB; a determination will be made regarding whether a new consent needs to be obtained from participants who provided consent, using a previously approved consent form. All patients will provide written informed consent before enrollment (S3 File). This study is registered at clinicaltrials.gov (NCT06336642), approved on 03/29/2024.

### Data management plans

Data will be entered in institutionally approved MD Anderson databases. All eligibility criteria must be satisfied prior to treatment initiation. All data collected will be used only for research purposes. Identifiers (name, medical record number, date of birth, and date of discharge) may be collected. Names and medical record numbers will be replaced by study numbers and dates of birth and death will be replaced by time intervals in the analytic files. Patient identifiers will be confidentially collected and securely maintained on a password-protected server located behind the institutional firewall. Access to identifiers will follow the MD Anderson IRB and MD Anderson information security rules and regulations. The master database file will be accessible only to the PI, approved co-investigators, and research staff designated on the delegation of authority log. Data will be shared on the Cancer Data Service repository.

### Data and safety monitoring committees

The Data Safety Monitoring Board (DSMB) is an officially constituted committee of MD Anderson that is designed to oversee the data safety monitoring of clinical trials. They are independent from the sponsor of this trial. The primary objectives of the DSMB are to 1) ensure that participants in a trial are protected; 2) ensure that participants’ interests are not made secondary to the interests of the scientific investigation; and 3) monitor all clinical trials that originate at MD Anderson or that are coordinated or analyzed by MD Anderson.

The DSMB is composed of scientists and statisticians from within and/or outside the institution, selected on the basis of their experience, reputation for objectivity, absence of conflicts of interest, and knowledge of good clinical trial methods. Individuals invited to serve on the DSMB disclose to the group chair any potential (real or perceived) conflicts of interest. These include professional interest, proprietary interest, and miscellaneous interest considerations. Further details on the MD Anderson DSMB may be found in in the supplement (S4 File).

### Protocol compliance

The research manager/supervisor will work with staff to monitor charts and protocol compliance by providing proper training with the PI. Protocol deviations will be documented in the deviation log and protocol violations/unanticipated problems will be reported to the IRB. Institutional Clinical Research Services (ICRS) also conducts audits randomly without prior notification to investigators in order to ensure adherence to established protocols.

### Statistical analysis

#### Sample size/accrual rate.

The primary endpoint, change of average dyspnea NRS intensity over 24 hours from baseline, is being examined in two separate cohorts, one of hypoxemic patients and another of non-hypoxemic patients. Therefore, we will use *p* < 0.025 to determine statistical significance. Linear mixed models (LMMs) with dyspnea intensity as the dependent variable and fixed terms for treatment, assessment time, and treatment × assessment time interaction will be created; intercept will be included as a random effect. If treatment × interaction is statistically significant with *p* < 0.025, we will use the model to test whether there is specifically a difference between arms at 24 hours. With 30 patients per arm in each cohort, we will have 80% power to detect a 1.2-point difference at 24 hours, assuming a standard deviation of 1.5 [14,29]. To account for patients enrolled in the trial who discontinue prior to treatment, we will recruit 75 patients per cohort in order to observe 60 patients (20% attrition, 150 patients total for 2 cohorts).

Given the short observation time and close monitoring in this trial, we expect few patients to have missing observations. As long as data are not missing at random, LMMs automatically handle missing data. Therefore, we will conduct analyses to examine whether participants who drop out of the study differ from those who do not and adjust for those covariates found to be related to missingness.

#### Analysis of primary endpoints.

We will assess the two primary outcomes: change from baseline NRS intensity at 24 hours in hypoxemic patients and change from baseline NRS intensity at 24 hours in non-hypoxemic patients using LMMs with fixed terms for treatment, assessment time, and treatment × assessment time interaction; intercept will be included as a random effect. If treatment × interaction is statistically significant with *p* < 0.025, we will use the model to test whether there is specifically a difference between arms at 24 hours. We will declare statistical significance if the two-sided *p*-value is 2.5% or less.

#### Analysis of secondary endpoints.

To assess patient outcomes over their 72-hour interventions, the same LMMs used for the primary objectives will be used to assess dyspnea intensity over 72 hours. Dyspnea unpleasantness, vital signs, symptom burden (ESAS), and quality of life (EQ-5D-5L) will also be examined using LMMs. Response (measured by improvement ≥ 1 in comparison to PDG and global assessment) and adverse events will be tabulated and tested for differences using chi-squared or Fisher’s exact tests as appropriate. Hospital mortality will be tested using a rate test. Length of hospital stay and length of ICU stay will be evaluated using 2-sample t-tests. We will evaluate patterns of use by tabulating order of modality and optimal settings and duration of each modality. Adverse event variables will combine the hypoxemic/non-hypoxemic patient cohorts, but all other analyses will analyze cohorts separately. These analyses will compare patients during the first 72 hours only. All testing in this aim will be 2-sided with 5% statistical significance. To avoid issues with multiple testing, results will be considered hypothesis generating rather than hypothesis testing.

To assess predictors of treatment response, we will examine if selected patient demographics (e.g., sex as a biological variable, age, race/ethnicity, baseline dyspnea intensity, obstructive/restrictive lung disease) and pattern of device use (e.g., duration of each modality) are associated with a treatment response to the SPOT-ON intervention. Multivariable logistic regression models will be used. These analyses, by definition, will be limited to patients randomized to receive SPOT-ON immediately and those who received SPOT-ON after 72 hours of enhanced usual care.

Finally, we will evaluate for any predictors related to preferences regarding oxygen delivery modalities using multinomial models.

An interim analysis for futility will be conducted once a patient cohort has information for half the patients enrolled to that cohort. The analyses will utilize the Lan-DeMets spending function with O’Brien-Fleming stopping boundaries. If the z-value after the interim analysis is 0.6 or higher, the trial will be stopped for futility. Enrollment will not be paused during interim analysis.

### Status and timeline of study

This study was approved by the MD Anderson IRB on 03/07/2024. We enrolled our first patient on 07/29/2024 and have enrolled 32 patients to date (8/27/2025). Recruitment is ongoing. Participant recruitment is expected to be completed December 2028, data collection is expected to be completed March 2029, and results are expected end of 2029.

### Dissemination plan

Scientific data generated as a result of this clinical trial will also be shared on the Cancer Data Service repository with sufficient quality to validate and replicate research findings. We expect to publish in peer-reviewed journals as well as present findings at national and international conferences.

## Discussion

This two-arm, parallel-group, partially blinded, waitlist-controlled, randomized trial will incorporate a personalized trial design to examine the optimal combination of oxygen and supportive therapies to palliate dyspnea in hospitalized patients with cancer. While HFNC and NIV are commonly deployed for respiratory failure in the inpatient setting, we will leverage their mechanisms of action beyond oxygenation and administer these therapies for dyspnea in a highly personalized manner aligned with patient preferences.

This clinical trial has incorporated multiple novel elements. First, to our knowledge, this will be the only clinical trial to specifically examine a RT-led intervention for the palliation of dyspnea. Second, we will examine how time-limited trials can be used to personalize dyspnea management by providing LFSO, HFNC, and/or NIV based on patient preference; akin to taste-testing, patients will have the opportunity to try multiple treatments and identify their treatment preferences. This approach contrasts with the more typical practice of patients being prescribed a treatment without learning about the alternatives. Third, we will test the effect of HFNC and NIV in both patients with mild/moderate hypoxemia and patients without hypoxemia, as these interventions are traditionally only offered to patients with severe hypoxemia. Fourth, we will assess Personalized Dyspnea Response as a novel outcome criterion [22,23]. Fifth, we will employ a personalized trial design with significant methodologic advantages (Table 3). Sixth, we will attempt to provide partial blinding by proper masking of research staff and patients through the incorporation of a waitlist control.

**Table 3:** Comparison of traditional and personalized clinical trial design.

Aspects	Traditional clinical trial design	Personalized clinical trial design
Patient selection	Highly restricted eligibility criteria; limited enrollment and generalizability	Broad eligibility criteria, as interventions will be personalized after enrollment; quicker enrollment and higher generalizability
Number of interventions	Single or few interventions; single modality	Multiple interventions; multimodal, with combinations based on patient preference
Nature of interventions	Fixed dose (all or none) and duration	Active titration of dose; time-limited trials to personalize treatments
Patient involvement	Passive participant subjected to preselected interventions	Active participant engaged in treatment choices
Expected outcomes	Small effect size	Larger effect size
Attrition	Higher, as treatments may not be tolerated	Potentially lower, as personalized therapies are flexible
Overall	Rigid design to examine individual interventions	Pragmatic, patient-centric design to examine multimodal interventions

From the clinical perspective, successful completion of the SPOT-ON trial may (1) lead to improved symptom control and quality of life in hospitalized patients with advanced cancer suffering from acute dyspnea; (2) inform the efficacy of a scalable, multimodal, personalized therapy for dyspnea management; (3) enhance clinicians’ understanding of patient preferences and adherence; and (4) inform novel models of respiratory care delivery. From the research perspective, successful demonstration of the effectiveness of time-limited trials and multimodal, personalized therapies to evaluate treatment response may open up further research opportunities to examine novel therapies for dyspnea, alleviate dyspnea in patients with non-cancer diagnoses (e.g., heart failure, chronic obstructive pulmonary disease), and develop analogous strategies for alleviating other distressing symptoms.

There are several limitations to our study design. First, although our team has extensive experience recruiting patients with advanced cancer and dyspnea [14,15,29–35], this population remains challenging to enroll due to the severity of illness, emotional distress, and multiple comorbidities. Second, patients may receive co-interventions during the trial as clinically indicated, which could complicate interpretation of the results; however, we will systematically document these co-interventions and adjust for them during analysis. Third, the collection of patient-reported outcomes presents logistical challenges, including the burden of gathering multiple data points across several timepoints, which may lead to missing data. Additionally, the inherently subjective nature of these outcomes may influence result interpretation.

Successful completion of this trial could yield invaluable insights into the use of oxygen and airflow modalities for dyspnea relief, the role of time-limited trials in facilitating rapid, individualized treatment decisions, the delivery of goal-concordant respiratory care based on patient preferences, and the potential advantages of personalized clinical trial design. These findings have the capacity to shift the paradigm of symptom management research and lay the groundwork for applying this innovative trial design to other symptoms and patient populations.

## Supporting information

S1 File SPIRIT checklist. (PDF)

S2 File Study protocol. (PDF)

S3 File Informed consent documents. (PDF)

S4 File Data safety monitoring board details. (PDF)
