# Supplementary material for: Structured Personalized Oxygen and Supportive Therapies for Dyspnea in Oncology (SPOT-ON): A personalized randomized clinical trial protocol
Source: PLoS One. 2025 Dec 2;20(12):e0336691. doi: 10.1371/journal.pone.0336691 (PMC12671826; doi:10.1371/journal.pone.0336691)
Supplement: S2 File — (PDF) [file pone.0336691.s002.pdf]

PROTOCOL TITLE:
Structured Personalized Oxygen and Supportive Therapies for Dyspnea in Oncology (SPOT-ON) Approach for Dyspnea Treatment in Cancer Patients: An R01 Randomized Clinical Trial

Protocol Number: 2023-0933

ClinicalTrials.gov Identifier: NCT06336642

Principal Investigator: Dr. David Hui

Co-Investigators:
Palliative Care: Dr. Eduardo Bruera, Dr. Minji Kim
Med Onc: Dr. Anne Tsao
Respiratory care: Gautam Sachdev, Saji Thomas, Dr. Nisha Rathi

Biostatisticians: Dr. Sanjay Shete, Bryan Fellman, MS, Ethan (Yi) Huang, MS

Version Number: 5

Date: 3/21/2025

TABLE OF CONTENTS

1 Introduction ... 6
1.1 Study Rationale ... 6
1.2 Background ... 7
1.3 Known Potential Risks ... 8
2 Objectives ... 8
2.1 Primary Objectives ... 8
2.2 Secondary Objectives ... 8
3 Study Design ... 9
4 Study Population ... 10
4.1 Eligibility Criteria ... 10
4.2 Exclusion Criteria ... 10
4.3 Screen Failures ... 10
4.4 Inclusion of Women and Minorities ... 11
5 Treatment Plan ... 11
5.1 Agent Administration ... 11
5.2 Preparation/Handling/Storage/Accountability ... 11
5.3 Dose Expansion Cohorts ... 12
5.4 General Concomitant Medication and Supportive Care Guidelines ... 12
5.5 Duration of Therapy ... 12
5.6 Duration of Follow-Up ... 12
5.7 Dosing Delays/Dose Modifications ... 12
6 Study Assessments and Procedures ... 13
6.1 Schedule of Activities (SoA) ... 13
7 Adverse Event, Serious Adverse Event (SAE) ... 18

8 Response Criteria 18
8.1 Data and Safety Monitoring Committees 18
8.2 Measurement of Effect 18
9 Statistical Considerations 18
9.1 Sample Size/Accrual Rate 19
9.2 Stratification Factors 19
9.3 Analysis of Primary Endpoints 19
9.4 Analysis of Secondary Endpoints 19
10 Study Oversight and Data Reporting Requirements 20
10.1 Data and Safety Monitoring 20
10.2 Clinical Trial Monitoring 20
10.3 Study Records Retention 20
10.4 Protocol Compliance 21
10.5 Consent Process and Documentation 21
11 Data Management and Sharing Plan 21
11.1 Data Type 21
11.2 Related Tools, Software and/or Code 22
11.3 Standards 22
11.4 Data Preservation, Access, and Associated Timelines 23
11.5 Access, Distribution, or Reuse Considerations 23
11.6 Data Collection and Management Responsibilities 24
11.7 Oversight of Data Management and Sharing 24
11.8 Incidental/Secondary Findings Disclosure Procedure 24
12 Statement of Compliance 24
13 References 25

LIST OF ABBREVIATIONS

AE	Adverse Event
ASCO	American Society of Clinical Oncology
CDS	Cancer Data Service
CI	Confidence Interval
CPAP	Continuous Positive Airway Pressure
CTCAE	Common Terminology Criteria for Adverse Events
dbGaP	Database of Genotypes and Phenotypes
DOI	Digital Online Identifier
ESAS	Edmonton Symptom Assessment System
EQ-5D-5L	EuroQol-5 Dimension-5 Level
FDA	Food and Drug Administration
HFNC	High-flow Nasal Canula
IRB	Institutional Review Board
LFSO	Low-flow Supplemental Oxygen
LMM	Linear Mixed Model
MCID	Minimal Clinically Important Difference
NIV	Non-invasive Ventilation
NRS	Numeric Rating Scale
PDG	Personalized Dyspnea Goal
PI	Principal Investigator
RC	Research Coordinator
RN	Registered Nurse
RT	Respiratory Therapist
SPOT-ON	Structured Personalized Oxygen and supportive Therapies for dyspnea in Oncology

STUDY SCHEMA

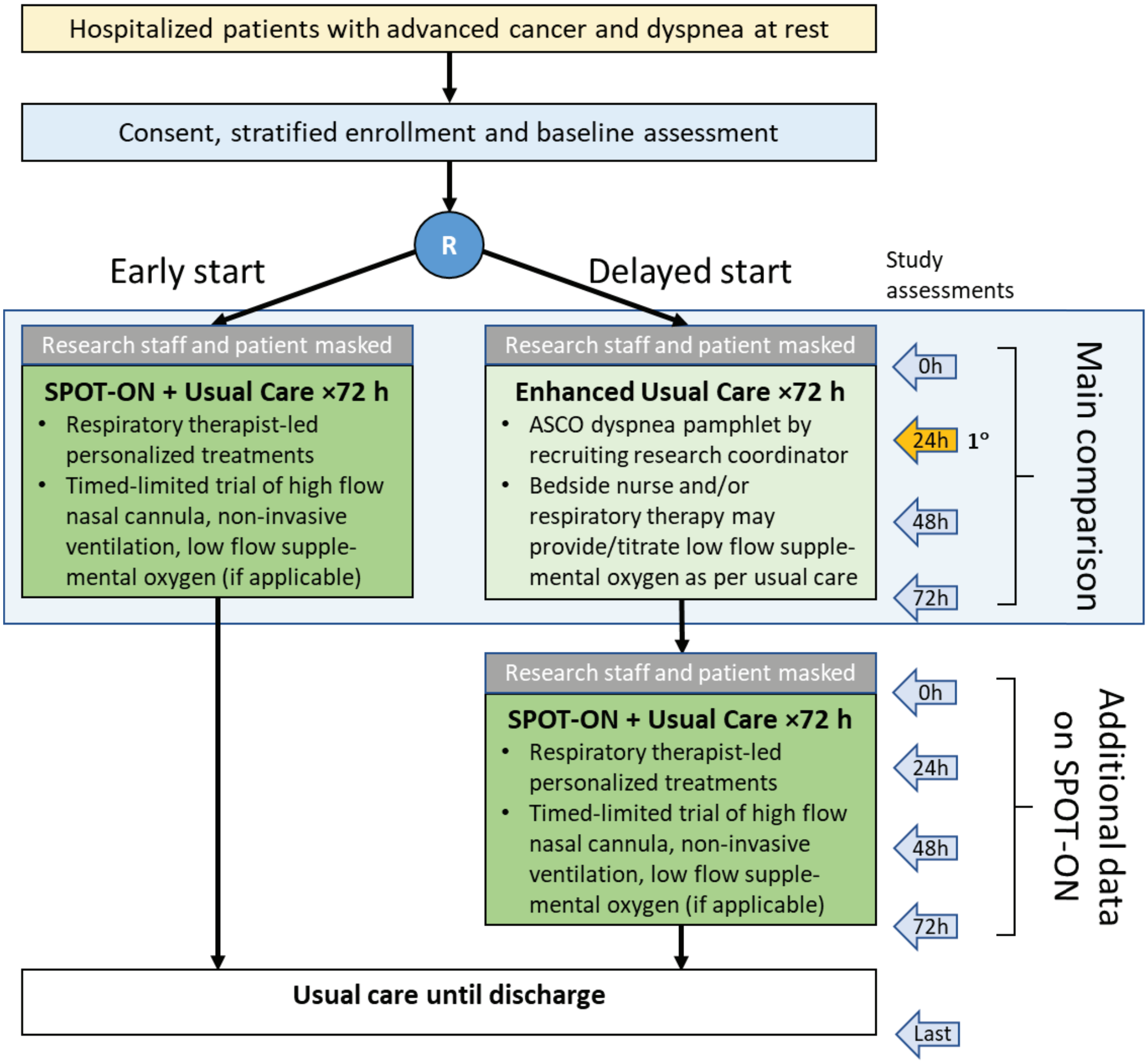

1 INTRODUCTION

1.1 Study Rationale

Dyspnea, the sensation of difficulty breathing, is a highly distressing symptom in patients with cancer.[1] Dyspnea is also highly prevalent, affecting up to 70% of patients with cancer,[2] particularly in those with intrathoracic malignancies and advanced disease.[3,4] Chronic dyspnea (i.e., > 4 weeks) is associated with decreased physical function, mood, quality of life, and survival.[5-7] Acute exacerbation of chronic dyspnea is the second most common symptom (34%) among the 4.5 million cancer patients presenting to the emergency room each year,[8,9] impacting the entire health care system. Indeed, dyspnea is reported in approximately 1 in 4 hospitalized patients (with or without cancer).[10]

Several large studies reported that 30–50% of patients with cancer experience refractory dyspnea in the last months of life, and the symptom increases in both frequency and intensity as death approaches.[11-13] Approximately half of these patients were hypoxemic, while the other half had normal oxygenation on room air.[14] Despite its high prevalence and negative impact, no therapies have been approved by the U.S. Food and Drug Administration (FDA) for dyspnea because of the paucity of high-quality clinical research. Therefore, dyspnea presents a major unmet need and public health concern. Multiple organizations, including the Institute of Medicine and the National Hospice and Palliative Nurses Association, identified dyspnea as a research priority.[15-17]

Parenchymal metastasis, lymphangitic carcinomatosis, airway obstruction, pleural effusion, pneumonia, pulmonary embolism, and atelectasis represent some common causes of dyspnea in the context of cancer.[17,18] The sensory cortex receives afferent input from various peripheral and central stimuli, generating the sensation of breathlessness.[1,19] Hypoxemia, defined as partial pressure of oxygen in the arterial blood (PaO_2) ≤ 60 mmHg or oxygen saturation in the arterial blood as detected by pulse oximeter (SpO_2) ≤ 90% on room air, may activate the O_2 sensitive chemoreceptors in the aortic and carotid bodies, leading to dyspnea. However, hypoxemia is only one of the mechanisms contributing to dyspnea; activation of mechanoreceptors, J receptors, and chest wall receptors may also result in dyspnea in patients with or without hypoxemia. In the advanced cancer setting, dyspnea is often multifactorial in nature with several co-existing etiologies. This calls for multimodal interventions to address dyspnea effectively.

Currently, there are several therapeutic options available to patients experiencing dyspnea. Non-pharmacologic therapies, including airflow interventions, supplemental oxygen, breathing techniques, meditation, and self-management techniques, are commonly used to treat dyspnea. Additionally, pharmacologic treatments for dyspnea, such as opioids, benzodiazepines, corticosteroids, and bronchodilators, can be recommended if non-pharmacologic strategies for palliating dyspnea are insufficient[17]. However, there is a wide range of patient responses to each of these dyspnea therapies, which adds undue stress onto patients if a prescribed treatment does not result in relieving their acute onset of dyspnea.

Many existing dyspnea clinical trials suffer from methodologic issues, including a lack of patient selection, a focus on chronic dyspnea (which is less responsive than acute dyspnea), examining a single intervention at a time, suboptimal intervention dosing and adherence, and under-powered sample size.[17] To overcome these concerns, we believe there are two fundamental insights that could potentially shift the paradigm of dyspnea research. First, because dyspnea is a subjective experience, the only way to know if an intervention works is for an individual to try it, necessitating a personalized approach. Based on our experience, acute dyspnea responds rapidly to interventions; short, time-limited therapeutic trials (5–30 minutes) would allow patients and clinicians to rapidly determine if the treatment is effective and to personalize dyspnea interventions in real time. Second, dyspnea is a complex symptom with afferent signals from chemoreceptors, mechanoreceptors, baroreceptors, and chest wall receptors, thus requiring a corresponding multimodal approach.[20,21] Based on these observations, we propose a Structured Personalized

Oxygen and supportive Therapies for dyspnea in Oncology (SPOT-ON) respiratory therapist (RT)-led intervention in which patients with no/mild/moderate hypoxemia would be offered a time-limited trial of high-flow nasal canula (HFNC), non-invasive ventilation (NIV), and low-flow supplemental oxygen (LFSO) to identify their optimal treatment(s), then implement and titrate the tailored therapy guided by patient preference.

1.2 Background

As a test of concept for time-limited trials, we conducted a four-period, four-intervention pilot crossover trial to compare the effect of high-flow oxygen, high-flow air, low-flow oxygen, and low-flow air for 10 minutes each in non-hypoxemic hospitalized patients with dyspnea (with variable washout).[22] Twenty-six patients were enrolled during the 17-month study period. Compared to low-flow air, high-flow oxygen (mean difference -1.24, 95% Confidence Interval (CI) -1.90, -0.57; $p < 0.001$) and high-flow air (mean difference -1.39, 95% CI -2.50, -0.37; $p < 0.001$) were both associated with a significant improvement in dyspnea, but not low-flow air (mean difference -0.44, 95% CI -1.11, 0.24; $p = 0.20$) (**Figure 1**). The treatments were well tolerated with no significant adverse events. Among patients who completed all treatment trials and reported their overall preference, 7 (54%) chose high-flow oxygen, 4 (31%) chose high-flow air, 1 (8%) chose low-flow oxygen and 1 (8%) chose low-flow air. Data from this study support (1) the rapid responsiveness of dyspnea to time-limited trials of oxygen and support interventions, (2) the potential benefit of the high-flow mechanism, (3) that patients were able to indicate their choice of treatment, (5) that many preferred a high flow rate, and (6) how RTs can effectively personalize device setting to optimize dyspnea management.

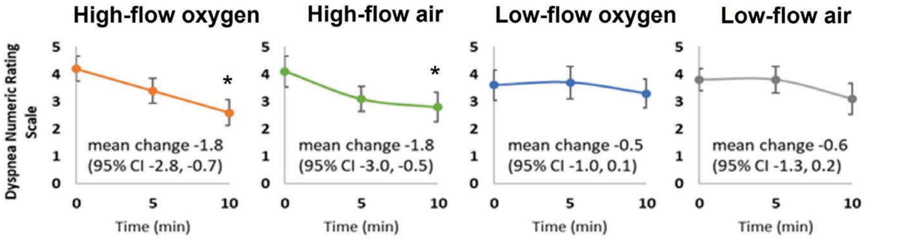

Figure 1. Rapid Relief of Dyspnea in Non-Hypoxemic Patients with High-Flow Nasal Cannula. In this randomized crossover trial, high-flow oxygen and high-flow air, but not low-flow oxygen and low-flow air, were associated with a significant improvement (asterisks; $p < 0.001$) in dyspnea within 10 minutes. CI: confidence interval. Error bars show standard error.

We conducted a two-period, two-intervention crossover trial to compare the efficacy of HFNC and NIV for 2 hours each in 30 hospitalized cancer patients with dyspnea and no/mild/moderate hypoxemia (**Figure 2**).[23] Twenty-six (93%) participants were on LFSO at baseline. RTs actively titrated the device settings to optimize comfort in the first five minutes. Significant improvements in the dyspnea numeric rating scale (NRS) were noted in both HFNC (mean improvement 1.9; 95% CI 0.4, 3.4; $p = 0.02$) and NIV (mean improvement 3.2; 95% CI 1.3, 5.1; $p = 0.004$). This study also included a variable washout period in which over 70% of patients reverted to baseline dyspnea level within 10 minutes once off intervention. In global symptom evaluation, 77% of patients on HFNC and 90% of patients on NIV reported that their dyspnea improved compared to baseline. Participants raised questions why these interventions were not more commonly available for their dyspnea. For example, one study

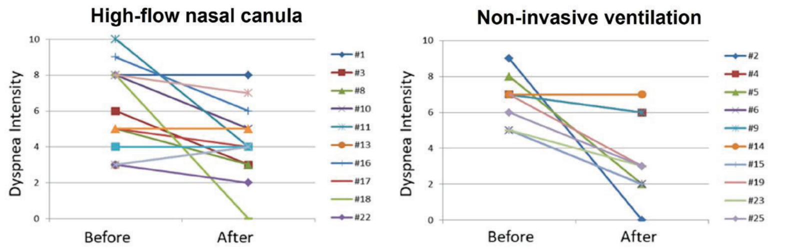

Figure 2. Reduction of Dyspnea with High-Flow Nasal Cannula and Non-Invasive Ventilation. In this randomized crossover trial, both high-flow nasal cannula (mean change 1.9, 95% CI 0.4-3.4; $p = 0.02$) and non-invasive ventilation (mean change 3.2, 95% CI 1.3-5.1; $p = 0.004$) were associated with a significant reduction of dyspnea numeric rating scale after 2 hours.

patient stated, "I liked the high flow for everyday use and NIV for times when I am particularly short of breath." This preliminary study highlights the efficacy of HFNC and NIV and patients' desire to have more flexibility in the use of these interventions.

1.3 Known Potential Risks

The aforementioned studies have found that HFNC and NIV to be well tolerated in patients with dyspnea with few adverse side effects.[24-27]

Known adverse effects related to supplemental oxygen use (applicable to HFNC, NIV, and LFSO) include the following:

- Dry or irritated nasal passages, mouth, or throat
- Eye irritation, such as dryness or conjunctivitis
- Decreased respiratory drive or respiratory depression, especially in patients with chronic hypercapnia or respiratory acidosis
- Absorption atelectasis, which can occur when oxygen displaces nitrogen in the alveoli, leading to alveolar collapse
- Increased risk of fire, especially if the patient is smoking or if oxygen leaks from the delivery device and makes contact with an ignition source
- Oxygen toxicity, which can lead to damage to the lungs, eyes, and nervous system, especially with prolonged exposure to high oxygen concentrations

Known adverse reactions specific to HFNC include the following:

- Nasal bleeding
- Infections, such as pneumonia

Known adverse reactions specific to NIV include the following:

- Discomfort from mask or skin irritation behind the ears or under the nose
- Difficulty tolerating the pressure support, such as air hunger, chest discomfort, or abdominal distension
- Increased work of breathing or airway resistance, which may lead to failure of NIV and need for invasive ventilation
- Worsening of the underlying respiratory or cardiac condition, such as increased oxygen requirement or pulmonary edema
- Infection, such as pneumonia or sinusitis

Risks posed by study questionnaires or the potential disclosure of confidential information are minimal, and the impact to subjects is expected to be negligible. Based on the preliminary data from our previous studies, we expect the personalized treatment options to be well tolerated.

2 OBJECTIVES

2.1 Primary Objectives

2.1.1 To determine the effect of SPOT-ON and Enhanced Usual Care on the change in **intensity of dyspnea** (NRS) between baseline and 24 h in **hypoxemic** hospitalized patients with cancer.

2.1.2 To determine the effect of SPOT-ON and Enhanced Usual Care on the change in **intensity of dyspnea** (NRS) between baseline and 24 h in **non-hypoxemic** hospitalized patients with cancer.

2.2 Secondary Objectives

2.2.1 To determine the effect of SPOT-ON and Enhanced Usual Care on **patient outcomes over 72 h**, including intensity of dyspnea (NRS), unpleasantness of dyspnea (NRS), dyspnea response,

vital signs, symptom burden, health-related quality of life (EQ-5D-5L), adverse events, patterns of device use, and hospital outcomes.

2.2.2 To identify **factors associated with dyspnea response** in the SPOT-ON intervention, including patient demographics, preferences, and level of usage of oxygen delivery modalities.

2.2.3 To identify **patient factors associated with their preferences** (after Phase II and Phase III) for each of the oxygen delivery modalities in the SPOT-ON intervention, such as patient demographics and dyspnea characteristics.

3 STUDY DESIGN

This is a partially blinded, two-arm, parallel-group, wait-list control, randomized trial of the SPOT-ON approach for dyspnea treatment in cancer patients (see Study Schema). This pragmatic, collaborative, patient-centered intervention is developed on the basis of our series of pilot randomized trials (Section 1.2), our extensive collaboration with respiratory therapy, and insights from co-investigators. It is designed to emulate elements of clinical practice to facilitate rapid adoption. Specifically, RTs are highly skilled in administering HFNC, NIV, and LFSO, conducting time-limited trials (typically for one modality only), adjusting device settings, and educating patients on oxygen and support modalities. The two arms of this study are the SPOT-ON intervention and the Enhanced Usual Care with wait-list control arm.

The SPOT-ON intervention arm will be completed in four phases (**Figure 3**). The first phase (Phase I) will consist of an orientation led by a blinded RT. The orientation will begin with an introduction to the patient and caregiver(s). The RT will review the patient's prior experience with oxygen and supportive therapies and verify any contraindications. Specifically, NIV and HFNC will not be offered to patients with fixed obstruction of upper airway (e.g., tumor), facial, upper airway, or upper gastrointestinal surgery within the past week, inability to protect the airway, or copious respiratory secretions. NIV will not be offered to patients with gastrointestinal bleed, bowel obstruction, or pneumothorax without intercostal drain insertion. An overview of the study intervention, blinding procedure, and study expectations will be discussed, as well as the patient's respiratory care goals. In total, the orientation should last approximately 20 minutes.

Phase II will proceed with the time-limited trials of the three available dyspnea treatment modalities: HFNC, NIV, and LFSO. The order of modalities tested will be randomized based on the patient picking between six unlabeled envelopes, each with a different order. Each modality will be tested for up to 10 minutes, with the goal of identifying the lowest possible setting to optimize dyspnea relief and overall comfort after optimization by the RT. Additionally, the RT will assess dyspnea, modality tolerance, vitals, and comfort in each time-limited trial. After all modalities have been tested, patient modality preference and treatment(s) of choice will be determined with the RT. Allowing for 10 minutes of initial set up, in total, this phase should last approximately 1 hour.

The third phase (Phase III) will implement the patient's dyspnea treatment preferences for a total intervention period of 72 hours, with time 0 set as immediately before start of Phase I. The RT will visit the patient twice daily (8 am and 2 pm) to provide personalized use of oxygen and support modalities. During these visits, patients may choose to adjust settings, switch between modalities, or discontinue any modality. Additional visits may be requested based on patient needs (up to 3x/day). During the study intervention, the RT will document the pattern of modality use over the 72 hours, time spent with the patient (i.e., RT time), and vitals. A dedicated research RT will be available from 8 am – 5 pm 7 days a week (2 will be specifically trained for this study). During after hours, other clinical RTs will follow the treatment plan outlined by the research RT. The study will continue until end of study intervention (72 h), discharge, patient deterioration (hemodynamic instability, delirium, respiratory failure requiring HFNC and NIV, or mechanical ventilation) or withdrawal – whichever occurs first.

The final phase of the SPOT-ON approach would involve weaning and/or stopping their oxygen modality. The patient may discuss further treatment options with their primary team.

Patients randomized into the Enhanced Usual Care with wait-list control arm receive a 20-minute educational session by the recruiting RC based on the American Society of Clinical Oncology (ASCO) Dyspnea Guideline patient education pamphlet (**Appendix A**). Over the next 72 hours, they will receive standard of care with treatments based on the attending teams' recommendations, including but not limited to, treatment of underlying causes, supportive care consultation, and other palliative measures. After 72 hours of usual care, patients in this group will start the aforementioned SPOT-ON intervention, and the research RT will be asked to start treatment provided they still meet study eligibility criteria (e.g., dyspnea intensity at rest of at least 4 on a 0 – 10-point NRS). The control group is carefully designed such that it reflects the current standard of care; enhanced usual care serves as an attention control; and the wait-list design provides patients with the opportunity to try SPOT-ON.

Thirty days after their intervention ends, patients will be followed up by research staff for AEs.

4 STUDY POPULATION

4.1 Eligibility Criteria

- Diagnosis of advanced cancer (metastatic, locally advanced, recurrent, or incurable).
- Age 18 or older.
- Admitted to a medical floor.
- Dyspnea intensity at rest of at least 4 on a 0–10-point NRS (where 0 = none, 10 = worst).
- Speak English or Spanish.

These eligibility criteria are intentionally broad with few exclusion criteria (see below) because patients with contraindications to a particular intervention will simply skip that treatment option as in routine clinical practice, which will help to maximize recruitment and generalizability.

4.2 Exclusion Criteria

- Hemodynamic instability requiring active Merit Team or ICU team involvement.
- Delirium as per clinical team's assessment in the Electronic Health Record (EHR).
- Severe hypoxemia (SpO_2 < 90% despite supplemental oxygen of up to 6 L/min).
- Continuous positive airway pressure (CPAP) use for obstructive sleep apnea, actively using >10 hours a day.
- Respiratory failure necessitating mechanical ventilation (i.e., HFNC or NIV), and planned thoracentesis within 72 hours of enrollment.
- Patients with known pregnancy.

4.3 Screen Failures

Participants who consented to participate in the clinical trial, but later found to not meet one or more eligibility criteria at the time of screening, are considered screen failures. These patients can be re-screened while they are in the hospital. A new accession number will also be added to account for each screen failure.

4.4 Inclusion of Women and Minorities

Both men and women of all races and ethnic groups are eligible for this trial. There will be no exclusions based on sex/gender or race/ethnicity. Patients with disabilities will be eligible for this study as long as they meet eligibility criteria. Patients will be required to speak English or Spanish. We will employ bilingual research staff and/or translators for verbal elements of the study (e.g., orientation, modality preferences, telephone assessments). All written assessments have already been validated for both English and Spanish (see Section 6.1).

The demographic profile of MD Anderson patients is 76% White, 9.4% Black or African American, 0.4% American Indian or Alaska Native, 0.1% Native Hawaiian or Other Pacific Islander, and 9.2% other. 14.3% of patients identify as Hispanic or Latino ethnicity. Our patients are slightly less diverse than the cancer patient population of Texas (68% non-Hispanic White, 17% Hispanic, 12% African American, and 2% Other race/ethnicity) because 48% of our patients come from outside of Texas, and the US cancer patient population is much less diverse than the Texas cancer population. Most of MD Anderson's patients have private insurance, Medicare, or both. Patient accrual from our hospital units will provide a sample that is ethnically and socio-economically representative of our patients with dyspnea. We expect to see a similar population represented in our study.

5 TREATMENT PLAN

5.1 Agent Administration

Treatment will be administered on an inpatient basis.

HFNC and NIV will be delivered using an FDA-approved Hamilton C1 ventilator with commercially available accessories, including nasal masks, facial masks, and nasal cannula (Hamilton Medical, Reno, Nevada).

HFNC and NIV have been FDA-approved for the delivery of gases in clinical settings.

For the HFNC modality, the settings will be as follows: initial setting (FiO_2 50%, 20 L/min, temperature 37°C), then RT will titrate FiO_2 (21–100%), flow rate (10–60L/min), and temperature (34–37°C) based on patient comfort and effect on dyspnea intensity.

For the NIV modality, the settings will be as follows: initial setting (FiO_2 50%, inspiratory pressure 10 cmH_2O, expiratory pressure 5 cmH_2O, nasal mask), then RT will titrate FiO_2 (21–100%), inspiratory pressure 8–12 cmH_2O, expiratory pressure 4–8 cmH_2O, and use of nasal/facial mask based on patient comfort and effect on dyspnea intensity.

For the LFSO modality, the settings will be as follows: initial setting (2 L/min or lowest rate to keep $SpO_2 \geq 90\%$), then RT will titrate FiO_2 (room air or oxygen) and flow rate (2–6 L/min) based on patient comfort and effect on dyspnea intensity.

5.2 Preparation/Handling/Storage/Accountability

FDA-approved Hamilton C1 ventilators are currently used throughout our institution for routine clinical care already, and installation and maintenance are performed per institutional policy governing medical devices. Clinical Engineering procedures consist of attaching preventive maintenance label and an asset tag on medical devices before use or installation in patient care. Clinical Engineering conducts and documents its activities in accordance with the Medical Equipment Management Plan and updates preventive maintenance labeling appropriately. This includes scheduled maintenance for medical devices, responding to Medical Device recalls, and responding to safety event reports. Documentation relating to the identity and qualifications of the individuals performing maintenance is maintained appropriately and according to institutional

policy. Oxygen administration will be titrated exclusively by the RT according to the patients' tolerance and preference.

5.3 Dose Expansion Cohorts

Not applicable

5.4 General Concomitant Medication and Supportive Care Guidelines

During the study period, patients will continue their usual treatments for dyspnea, including opioids, supplemental oxygen, bronchodilators, and supportive/palliative care consultation. These treatments will be documented and adjusted for in the analysis.

5.5 Duration of Therapy

In the absence of treatment delays due to adverse event(s), treatment may continue for the scheduled 72 hours or until one of the following criteria applies:

- Patient decides to withdraw from the study
- Unacceptable adverse event(s)
- Worsening on clinical status – i.e., meeting exclusion criteria
- General or specific changes in the patient's condition render the patient unacceptable for further treatment in the judgment of the investigator

The reason(s) for protocol therapy discontinuation, the reason(s) for study removal, and the corresponding dates will be documented.

5.6 Duration of Follow-Up

Patients will be followed for approximately 30 days after removal from study or until death, whichever occurs first. Recruiting Coordinator will follow-up on any ongoing adverse events at end of treatment. New adverse events, specifically those listed within the consent form will be reviewed at the follow up period. Adverse Event document (Appendix K) is not required at the 30 day follow up period. Patients removed from study for unacceptable adverse event(s) will be followed until resolution or stabilization of the adverse event.

5.7 Dosing Delays/Dose Modifications

The FDA-approved Hamilton-C1 ventilator (Hamilton Medical, Reno, Nevada) is a versatile and feature-rich ventilator packaged in a compact size that can switch between invasive or NIV and HFNC without changing the device or the breathing circuit. The integrated, high-performance turbine enables the Hamilton-C1 to be completely independent from compressed air for maximum mobility. Capabilities include: full life-support 24/7; NIV with peak flow rate up to 260 L/min and leak compensation up to 120 L/min; HFNC therapy with flow rates from 2–100 L/min for adult/pediatric patients; invasive modes: adaptive volume-controlled modes, pressure-controlled modes (including biphasic modes), and volume support mode; individualized, lung-protective ventilation modes; adaptive support ventilation (ASV), which adapts ventilation breath-by-breath, 24 hours a day, from intubation to extubation, and continuous adjustment of respiratory rate, tidal volume, and inspiratory pressure depending on the patient's lung mechanics and effort. This device is currently used throughout our institution for routine clinical care already, and our RTs are thus highly familiar with this device.

The Hamilton C1 ventilator will be used to deliver HFNC and NIV. We will use commercially available accessories including nasal masks, facial masks, and nasal cannula (Hamilton Medical, Reno, Nevada). Oxygen titration will be optimized per individual patient by the RT for both HFNC

and NIV dyspnea relief modalities. For patients with hypoxemia (i.e., $PaO_2 \leq 60$ mmHg or $SaO_2 \leq$ 90% on room air), the RT will always ensure adequate oxygenation is provided to ensure SpO_2 is between 92–97%. For patients without hypoxemia, the RT will be able to use medical air or supplemental oxygen. During time-limited trials, patients will have the opportunity to try each LFSO, HFNC and NIV between 5–30 minutes at a time. During the 72-hour SPOT-ON intervention phase, the RT will work with the patient to personalize device settings (titrate up or down depending on dyspnea, tolerance, overall comfort, and patient preference, see Section 5.1). Patients will also have the ability to decide how often they use each modality. The device settings and pattern of use will be recorded (**Figure 3**).

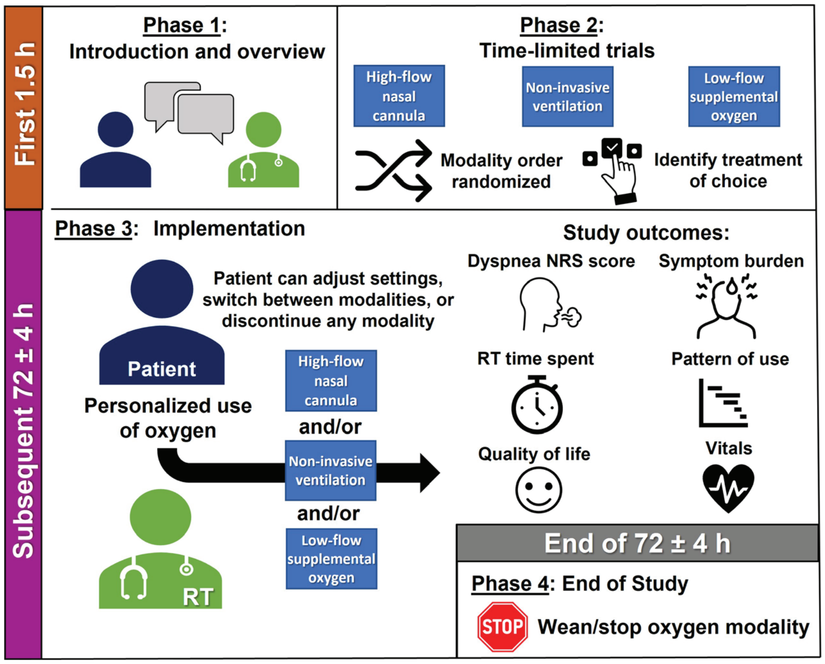

Figure 3. SPOT-ON Study Logistics. In this intervention, a respiratory therapist will identify preferred dyspnea treatment choices with individual patients based on time-limited trials and offer personalized use of oxygen using the specified modalities over the remaining 72 ± 4 hours. Acronyms: NRS, Numeric Rating Scale; RT, Respiratory Therapist.

6 STUDY ASSESSMENTS AND PROCEDURES

6.1 Schedule of Activities (SoA)

Recruitment and Enrollment: Patients admitted to MD Anderson will be screened systematically on the above eligibility criteria based on a combination of electronic health records and in-person assessments. If patients are eligible and interested, we will ask them to sign an informed consent after obtaining permission from the attending team during the same hospital visit. The time between patient screening and patient enrollment is expected to range from one hour to three days. To improve recruitment/retention, we will offer participants a $50 gift card upon completion of the study to compensate them for their time and effort. No competing studies will interfere with enrollment.

Training and Intervention Fidelity: All research staff, including the research RT and study coordinators, will undergo an 8-hour orientation before study activation to discuss (1) dyspnea, (2) menu of interventions, (3) communication skills, (4) study procedures including blinding (Table 1), and (5) pitfalls and solutions. They will be provided with a research manual that includes standard operating procedures. Research staff will attend weekly Principal Investigator (PI) meetings. All clinical RTs (other than research RTs) will receive a 30-minute orientation, so they are aware of

the study intervention and procedures. The PI or designate will assess intervention fidelity with direct observation using a standardized checklist (first 3 consecutive patients, then randomly every 10 patients).

While patients will know when they are participating in the SPOT-ON intervention, we will state in the consent document that we will be randomly allocating a time to start SPOT-ON but not disclose the specific timing of the two study groups (i.e., immediately vs. 72 hours later). This would allow us to blind the patients from knowing their treatment group assignment to minimize bias, without affecting their care because both groups will have access to SPOT-ON. The timing of the study groups will be determined at random by computer using the Clinical Trial Conduct website developed by the Department of Biostatistics. The 20-minute educational session (described below) that patients assigned to enhanced usual care will receive immediately after enrollment will partially blind them to the actual intervention. This masking is particularly appropriate when the risk of not disclosing the exact timing is considered minimal.

We will assess the intactness of masking by asking RTs, blinded research coordinators (RC), and patients to guess their group assignment at 72 ± 4 hours.

Table 1. Blinding Procedures in Wait-List Control Trial

Individuals	Blinding Status	Roles and responsibilities
Recruiting research coordinator (not blinded)	Unblinded but will not be involved in intervention delivery study nor outcome assessments	Obtain assignment from randomization website; conduct baseline assessments only; notify blinded research coordinators and research respiratory therapists to start their parts; provide education for enhanced usual care group. For patients randomized to Delayed Start, all assessments conducted by the blinded coordinator during Enhanced Usual Care Phase, will subsequently be conducted by the Recruiting Coordinator during the SPOT-ON + Usual Care Phase
Blinded research coordinator (blinded)	Unaware what treatment intervention the patient is receiving and when patient enrolled onto trial.	Conduct all key study assessments for 3 d, using telephone to maintain blinding (SPOT-ON or usual care, intervention group or wait-list control)
Research respiratory therapist (RT)	Unaware if patient is on wait-list control or not<br>Masked to timing of enrollment	Conduct focused respiratory therapy and assessments during SPOT-ON intervention
Patient (partially blinded)	Will not be aware of the two different study groups in the study (difference in start time)	Work with research RTs to complete intervention and research coordinators to complete assessments
Principal investigator (not blinded)	Unblinded but will not be involved in intervention delivery nor outcome assessments	Overall training and monitoring of study<br>Monitor SPOT-ON fidelity

Educational Session: Immediately after enrollment, patients assigned to the wait-list control group will receive a 20-minute educational session by the recruiting RC based on the ASCO Dyspnea Guideline patient education pamphlet (**Appendix A**). Patients assigned to the SPOT-ON group will receive a similar 20-minute session that covers 1) an introduction to the patient and caregiver(s); 2) a review of the patient's prior experience with oxygen, support interventions, and any known contraindications; 3) the study intervention overview and blinding procedures; 4) setting study expectations; and 5) discussing patient's goals for respiratory care.

Time-Limited Trials: Patients beginning the SPOT-ON protocol will be led through time-limited trials of the three available dyspnea treatment modalities: HFNC, NIV, and LFSO. The order of

modalities tested will be randomized based on the patient picking between six unlabeled envelopes, each with a different order. Each modality will be tested for up to 10 minutes, with the goal of identifying the lowest possible setting to optimize dyspnea relief and overall comfort after optimization by the RT. Additionally, the RT will assess dyspnea, modality tolerance, vitals, and comfort each time-limited trial, with the assistance of the unblinded recruiting coordinator. After all modalities have been tested, patient modality preference and treatment(s) of choice will be determined with the RT.

Preferred Interventions (SPOT-ON) and/or Enhanced Usual Care Interventions: For 72 hours, enrolled patients will receive supportive dyspnea care by an RT. The manner of care will be determined by which group they are assigned to. Patients in the SPOT-ON intervention will have determined which dyspnea treatment option they would prefer to use, with the option to switch modalities during the 72 hours. Patients in the Enhanced Usual Care wait-list group will receive supportive care at the discretion of the attending clinician, as is standard practice. All SPOT-ON interventions will be monitored by the RT, who will titrate the oxygen delivery as needed.

Table 2. Study Assessments

Assessment	Patient time	Baseline (Immed-iately before Phase I)	Time-limited Trials (Phase II)	SPOT-ON or Enhanced Usual Care Delivery (Phase III)		
				24 ± 4 h	48 ± 4 h	72 ± 4 h
Baseline assessments (demographics, spirometry, highest inspiratory flow during tidal breathing, maximal inspiratory pressure) [Recruiting RC] (**Appendix B**)	20 minutes	✓				
Personalized dyspnea goal [Recruiting RC] (**Appendix C**)	1 minute	✓				
Time-limited trial assessments (modality order, dyspnea NRS, vital signs, comfort/tolerance, modality preference) [RT] and/or [Recruiting RC] (**Appendix D**)	~15 minutes		✓			
Dyspnea NRS score (primary outcome: intensity; secondary: unpleasantness) [Blinded RC] and/or [Recruiting RC] (**Appendix E**)	1 minute	✓		✓	✓	✓
Symptom burden (ESAS) [Blinded RC] and/or [Recruiting RC] (**Appendix F**)	< 3 minutes	✓		✓	✓	✓
Health-related quality of life (EQ-5D-5L) [Blinded RC] and/or [Recruiting RC] (**Appendix G**)	< 2 minutes	✓		✓	✓	✓
Medications/co-interventions [Recruiting RC] (**Appendix H**)	-	✓		✓	✓	✓
Vital signs [Recruiting RC] (**Appendix I**)	< 5 minutes	✓		Every 8 h	Every 8 h	Every 8 h
Global assessment (< 1 minute) [Blinded RC] and/or [Recruiting RC] (**Appendix J**)	1 minute			✓	✓	✓
Adverse events (CTCAE v5.0) [Recruiting RC] (**Appendix K**)	< 2 minutes			✓	✓	✓
Pattern of device use [RT]or [Recruiting RC] (**Appendix L**)	-		✓	With each session of device use		
Hospital outcomes[Recruiting RC] (**Appendix M**)	-					✓
Blinding [Blinded RC] (**Appendix N)**				✓		
mMRC Dyspnea Scale [Recruiting RC] **(Appendix O)**	1 minute	✓				

Abbreviations: NRS, Numerical Rating Scale; CTCAE, Common Terminology Criteria for Adverse Events; EQ-5D-5L, EuroQol-5 Dimension-5 Level; ESAS, Edmonton Symptom Assessment System; RC, research coordinator; RT, respiratory therapist; RN, registered nurse
Note: for patients on the wait-list control who completed the first 3 days of usual care, they will have another 3 days of assessments as if they were receiving SPOT-ON upfront. They would provide valuable information on SPOT-ON intervention.
Timing: If a patient is unable or unwilling to complete assessments or interventions within the time-period specified, timing may be delayed at the discretion of the Investigator.

- **Administration of questionnaires or other instruments.** Our research staff are trained to facilitate patients' completion of the questionnaires, while being sensitive and respecting patients' choices if they refuse to answer any questions. We have statistical plans to handle missing data.
- **Baseline assessments.** Prior to randomization into the two interventions, patients will undergo the following assessments performed by an unblinded recruiting RC (**Appendix B**):
    - Patient demographics
    - Cardiopulmonary comorbidities
    - Karnofsky performance status
    - Medication use
    - Spirometry
    - Maximal inspiratory pressure
    - Highest inspiratory flow during tidal breathing
- **Personalized dyspnea score** will be established at the baseline of the study, prior to randomization into the two interventions. Personalized dyspnea response is defined as dyspnea intensity NRS ≤ Personalized Dyspnea Goal (PDG).[28,29] An unblinded recruiting RC will assess PDG at enrollment by asking "At what level of intensity would you feel comfortable, on a scale of 0 to 10, where 0 = no shortness of breath and 10 = worst possible?" (**Appendix C**).
- **Time-limited Trials.** Oxygen delivery device order will be notated by the RT and/or the unblinded recruiting RC. If any devices are not given, the specific reason will be recorded. During time-limited trials, we will measure vital signs including heart rate, respiratory rate, and blood pressure before and after each modality among patients randomized to the SPOT-ON intervention. Dyspnea before and after each modality will also be assessed. Patient comfort/tolerance and global assessment will also be assessed after each modality tested. Modality preference will be recorded after time-limited trials have concluded in person by the blinded RT. Patients will be asked to rank the modalities they tested based on their overall comfort and tolerance and to indicate the modalities they would prefer to use for different instances of dyspnea (i.e., acute attacks vs. chronic) and if they would prefer not to use a particular modality. Additionally, they will be asked to indicate how they would like to use each of the devices over the next three days for maximum comfort (**Appendix D**).
- **Dyspnea intensity and unpleasantness** will be recorded at the baseline of the study, prior to randomization into the two interventions, by an unblinded recruiting RC by asking the patients to rate their dyspnea intensity and unpleasantness (two separate outcomes/questions) over the past 24 hours using the dyspnea NRS (i.e., ranging from 0 to 10). Additionally, a blinded RC, via telephone, will assess dyspnea intensity and unpleasantness in both the SPOT-ON and Enhanced Usual Care interventions at baseline (immediately before Phase I), 24 ± 4, 48 ± 4, and 72 ± 4 hours (**Appendix E**). We will also assess the worst and lowest dyspnea intensity over past 24 h and dyspnea intensity now. For patients randomized to Delayed Start, assessment will subsequently be conducted by the unblinded recruiting RC during the SPOT-ON + Usual Care Phase.

  - We will ensure that each patient understands the differences between intensity and unpleasantness of breathlessness by providing clear instructions as follows: “Intensity refers to the pure level or magnitude of the sensation. It is like a physical measure: for example, ‘how much do you weigh in pounds?’ Intensity does not contain any pleasantness or unpleasantness, like or dislike, or measure of how terrifying the experience is to you. Unpleasantness describes how much you like or dislike something or feel terrified by it. High unpleasantness indicates that your breathing feels very bad or terrifying regardless of whether the intensity is high or low.”

- **Edmonton Symptom Assessment System (ESAS**) questionnaire to assess **symptom burden** will be administered by a blinded RC, via telephone, to patients in both the SPOT-ON and Enhanced Usual Care interventions at the following time points: baseline (immediately before Phase I), 24 ± 4, 48 ± 4, and 72 ± 4 hours (**Appendix F**). The ESAS has been validated in English and Spanish.[30,31] For patients randomized to Delayed Start, ESAS will be conducted by the Recruiting Coordinator during the SPOT-ON + Usual Care Phase.

- **EuroQol-5 Dimension-5 Level (EQ-5D-5L)** questionnaire to assess **health-related quality of life** will be administered by a blinded RC, via telephone, to patients in both the SPOT-ON and Enhanced Usual Care interventions at the following time points: baseline (immediately before Phase I), 24 ± 4, 48 ± 4, and 72 ± 4 hours (**Appendix G**). EQ-5D-5L has been validated in English and Spanish.[32-34] For patients randomized to Delayed Start, (EQ-5D-5L) will be conducted by the Recruiting Coordinator during the SPOT-ON + Usual Care Phase.

- **Medications/co-interventions.** Medications and co-interventions outside of the scope of the SPOT-ON intervention will be documented at baseline (immediately before Phase I) then daily during Phase III by the Recruiting Coordinator (**Appendix H**).

- **Vital signs.** During the intervention stage, we will measure vital signs (i.e., heart rate, respiratory rate, and blood pressure) at baseline (immediately before Phase I) and every 8 hours in both the SPOT-ON and Enhanced Usual Care wait-list group. We will import additional vital sign data from Epic during the hospital stay if already collected as per routine clinical practice (**Appendix I**). If using Epic as a source of documentation for Vital Signs, Appendix I. will not be required.

- **Global assessment.** Global impression of change will be assessed by a blinded RC, via telephone, to directly compare the level of dyspnea before and after the study intervention by asking patients if they felt their dyspnea is better (a little better, somewhat better, moderately better, a good deal better, a great deal better, very great deal better), about the same, or worse (a little worse, somewhat worse, moderately worse, a good deal worse, a great deal worse, very great deal worse) after 24 ± 4, 48 ± 4, and 72 ± 4 hours on the SPOT-ON and Enhanced Usual Care interventions.[35-37] This will be referred to as the Global Assessment (**Appendix J**).

- **Assessment of adverse events.** Adverse events will be noted by the recruiting coordinator using the Common Terminology Criteria for Adverse Events (CTCAE) v5.0 assessment in both the SPOT-ON and Enhanced Usual Care interventions at the following time points: 24 ± 4, 48 ± 4, and 72 ± 4 hours. Patients will also be followed for 30 days after end of treatment, or until death, whichever occurs first. Patients removed from study for unacceptable adverse event(s) will be followed until resolution or stabilization of the adverse event (**Appendix K**).

- **Pattern of device use** will be logged at the end of Phase II and daily by the RT during Phase III. This will additionally be logged by the recruiting coordinator during the Enhanced Usual Care Phase. Device start and end times, in addition to device settings, will be noted (**Appendix L**).

- **Hospital outcomes**, including length of hospital stay, length of ICU admission, in-patient palliative care consultation, hospital mortality, and discharge location, will be recorded by the non-blinded RC after discharge or end of study, whichever occurs first. (**Appendix M**).

- **Blinding** will be assessed by having patients and blinded RCs guess which treatment intervention they received first (i.e., SPOT-ON or waitlist) at 24 ± 4 hours, only at the primary end point, by the blinded RC.(**Appendix N**).
- **Modified Medical Research Council (mMRC) Dyspnea Scale** will be used to evaluate the impact of dyspnea on daily life. This will be recorded at the baseline of the study, prior to randomization into the two interventions, by an unblinded recruiting RC. **(Appendix O)**

7 ADVERSE EVENT, SERIOUS ADVERSE EVENT (SAE)

An Adverse Event is any untoward medical event that occurs during participating in clinical research.

Adverse events will be captured and graded using the National Cancer Institute's Common Toxicity Criteria for Adverse Events (CTCAE) v. 5.0 (or latest version) and will include the event term, grading, duration and attribution. Attribution to the study agent or intervention will be determined by the PI, Co-PI, or treating physician.

Only Serious Adverse Events considered at least possibly related to study treatment, subsequent to starting SPOT-ON therapy, will be reported within 10 working days after notification of the event. All Fatal Adverse Events will be promptly reported according to institutional standards. The study period during which all AEs and SAEs must be reported begins from the time of the first protocol-specific intervention and initiation of study treatment and ends 30 days following the last administration of study treatment or study discontinuation/termination, whichever is earlier.

AEs of Special Interest (AESIs) are a subset of events to monitor of scientific and medical concern specific to the investigational product, for which ongoing monitoring and rapid communication by the Investigator to the Sponsor is required. Such an event might require further investigation in order to characterize and understand it. Depending on the nature of the event, rapid communication by the trial Sponsor to other parties (e.g., Regulatory Authorities) may also be warranted.

8 RESPONSE CRITERIA

8.1 Data and Safety Monitoring Committees

MD Anderson's Data and Safety Monitoring Committees are responsible for monitoring all investigator-initiated Pilot, Phase I, I/II, II single arm, as well as randomized Phase II or higher clinical trials. This trial will adhere to institutional data safety monitoring plans.

8.2 Measurement of Effect

The Minimal Clinically Important Difference (MCID) for dyspnea intensity and unpleasantness is a decrease 1 point for improvement on the NRS.[38] However, because the MCID cutoff values for response apply only to group averages instead of individual patients, it is difficult to judge whether a patient has achieved a response. Furthermore, with the MCID approach, patients with higher baseline symptom intensity were often more likely to report a response. Therefore, dyspnea intensity NRS pre- and post-intervention will also be compared to the patient's PDG, established prior to the time-limited trials phase of the study; this will allow us to assess dyspnea response on the individual level.

9 STATISTICAL CONSIDERATIONS

9.1 Sample Size/Accrual Rate

We anticipate enrolling approximately 150 patients (75 in the hypoxemic cohort and 75 in the non-hypoxemic cohort) in order to observe 120 after attrition, with an expected accrual rate of enrolling 3 patients per month. Based on our preliminary data,[14] we expect a similar proportion of patients with and without hypoxemia to be eligible for this study.

The primary endpoint, change of average dyspnea NRS intensity over 24 h from baseline, is being examined in two separate cohorts, one of hypoxemic patients and another of non-hypoxemic patients. Therefore, we will use $p < 0.025$ to determine statistical significance. Linear mixed models (LMMs) with dyspnea intensity as the dependent variable and fixed terms for treatment, assessment time, and treatment × assessment time interaction will be created; intercept will be included as a random effect. If treatment x interaction is statistically significant with $p < 0.025$, we will use the model to test whether there is specifically a difference between arms at 24 hours. With 30 patients per arm in each cohort, we will have 80% power to detect a 1.2 point difference at 24 hours, assuming a standard deviation of 1.5.[23,39] To account for patients enrolled in the trial but who discontinue prior to treatment, we will recruit 75 patients per cohort in order to observe 60 (20% attrition, 150 patients total for 2 cohorts).

Given the short observation time and the close monitoring, we expect few patients to have missing observations. As long as data are not missing at random, LMMs automatically handle missing data. Therefore, we will conduct analyses to examine whether participants who drop out of the study differ from those who do not and adjust for those covariates found to be related to missingness.

9.2 Stratification Factors

Randomization will only be stratified by baseline dyspnea intensity at rest (≤ 6/10 vs. ≥ 7/10); both the hypoxemic and non-hypoxemic cohorts will utilize these factors.

9.3 Analysis of Primary Endpoints

We will assess the 2 primary objectives, change from baseline NRS intensity at 24 hours in hypoxemic patients (Section 2.1.1) and change from baseline NRS intensity at 24 hours in non-hypoxemic patients (Section 2.1.2), using LMMs with fixed terms for treatment, assessment time, and treatment × assessment time interaction; intercept will be included as a random effect. If treatment x interaction is statistically significant with $p < 0.025$, we will use the model to test whether there is specifically a difference between arms at 24 hours. We will declare statistical significance if two-sided *p*-value is 2.5% or less.

9.4 Analysis of Secondary Endpoints

To assess patient outcomes over their 72-hour interventions, the same models used for the primary objectives (linear mixed models, LMMs) will be used to assess dyspnea intensity over 72 hours. Dyspnea unpleasantness, vital signs, symptom burden (ESAS), and quality of life (EQ-5D-5L) will also be examined using LMMs. Response, as measured by improvement ≥ 1, by comparison to personalized dyspnea goal and with global assessment, and adverse events will be tabulated and tested for differences using chi-squared or Fisher's exact tests, as appropriate. Hospital mortality will be tested using a rate test. Length of hospital stay and length of ICU stay will be evaluated using 2-sample t-tests. We will evaluate patterns of use by tabulating order of modality and optimal settings and duration of each modality. Adverse event variables will combine the hypoxemic/non-hypoxemic patient cohorts, but all other analyses will analyze cohorts separately. These analyses will compare patients during the first 72 hours only. All testing in this aim will be 2-sided with 5% statistical significance. To avoid issues with multiple testing, results will be considered hypothesis generating rather than hypothesis testing.

To assess predictors of treatment response, we will examine if selected patient demographics (e.g., sex as a biological variable, age, race/ethnicity, baseline dyspnea intensity, obstructive/restrictive lung disease) and pattern of device use (e.g., duration of each modality) are associated with a treatment response to the SPOT-ON intervention. Multivariable logistic regression models will be used. These analyses, by definition, will be limited to patients randomized to SPOT-ON and those who received SPOT-ON after being on enhanced usual care.

Finally, we will evaluate for any predictors related to preferences regarding oxygen delivery modalities using multinomial models.

An interim analysis for futility will be conducted once a patient cohort has information for half the patients enrolled to that cohort. The analyses will utilize the Lan-DeMets spending function with O'Brien-Fleming stopping boundaries. If the z-value after the interim analysis is 0.6 or higher, the trial will be stopped for futility. Enrollment will not be paused during interim analysis.

10 STUDY OVERSIGHT AND DATA REPORTING REQUIREMENTS

This protocol is monitored at several levels, as described elsewhere in this section. The Protocol Principal Investigator is responsible for monitoring the conduct and progress of the clinical trial, including the ongoing review of accrual, patient-specific clinical data, and routine and serious adverse events; reporting of expedited adverse events; and accumulation of reported adverse events from other trials testing the same interventions. The Protocol Principal Investigator and statistician always have access to the data.

All Study Investigators at participating sites who register/enroll patients on a given protocol are responsible for timely submission of data via the mechanism described elsewhere in this section. All studies are also reviewed in accordance with the enrolling institution's data safety monitoring plan.

10.1 Data and Safety Monitoring

Data and Safety Monitoring is the process for reviewing data collected as research progresses to ensure the continued safety of current and future participants as well as the scientific validity and integrity of the research. Studies conducted at MD Anderson will follow the DSMP that has been approved by the NCI.

The Principal Investigator is ultimately responsible for the conduct and monitoring of all aspects of the study on an ongoing basis. The Principal Investigator will provide an annual review and report of the study, including all adverse events, accrual information, efficacy and response data, along with overall study progress and continuation plans to MD Anderson's Data and Safety Monitoring Committees responsible for study oversight.

10.2 Clinical Trial Monitoring

Regular monitoring of trial conduct will be conducted to ensure that the rights and well-being of trial participants are protected, that the reported trial data are accurate, complete, and verifiable, and that the conduct of the trial is in compliance with the currently approved protocol/amendment(s), with ICH GCP, and with applicable regulatory requirement(s).

10.3 Study Records Retention

Essential/Source Documents may be maintained in electronic or via paper records. Electronic records must be maintained on an MD Anderson secured server accessed only by authorized

research staff. Paper records must be maintained in a lockable room or cabinet accessed only by authorized research staff and must be made available in the event of an audit/inspection.

A plan for continued storage of Essential/Source Documents consistent with protocol requirements, applicable regulations, Clinical Research contracts, and the Medical Records Policy (MD Institutional Policy CLN0554) will be followed.

10.4 Protocol Compliance

Research manager/supervisor will work with staff to monitor charts and protocol compliance by providing proper training with the PI. Additional random institutional audits will also ensure compliance. Protocol deviations will be documented in the deviation log and protocol violations/unanticipated problems will be reported to IRB.

10.5 Consent Process and Documentation

Please check one of the following:

☒ This protocol will follow the SOP 04_Informed Consent Process. SOP 04 has been read by the research staff and investigators.

☐ This protocol will follow SOP 04_Informed Consent Process with the following changes: . SOP 04 has been read by the research staff and investigators.

Please indicate what type of consent process will be used (check all that apply):

☒ Remote consent

☒ In-person consent

☐ Waiver of consent

☐ Waiver of written documentation of consent

11 DATA MANAGEMENT AND SHARING PLAN

11.1 Data Type

- **Types and amount of scientific data expected to be generated in the project.** Demographic, clinical, and quantitative data will be acquired from 150 participants, as further described in research narrative. Datasets generated in the project will be as follows:

  - Dataset #1: Demographics
    - 1 time per 150 participants
  - Dataset #2: Baseline respiratory function (highest inspiratory flow during tidal breathing, spirometry, vital signs, maximal inspiratory pressure)
    - 1 time per 150 participants
  - Dataset #3: Dyspnea NRS score
    - 5 timepoints per 150 participants
  - Dataset #4: Participants Personalized Dyspnea Score
    - 1 timepoint per 150 participants
  - Dataset #5: Vital signs
    - Up to 9 timepoints per 150 participants
  - Dataset #6: Symptom burden (ESAS-Edmonton Symptom Assessment System)
    - 4 timepoints per 150 participants

- Dataset #7: Quality of life (EQ-5D-5L-EuroQol-5 Dimension-5 Level)
    - 4 timepoints per 150 participants
- Dataset #8: Adverse events (CTCAE v5.0- Common Terminology Criteria for Adverse Events)
    - 4 timepoints per 150 participants
- Dataset #9: Pattern of device use
    - Minimum of 4 timepoints per 150 participants
- Dataset #10: Global assessment
    - 3 times per 150 participants
- Dataset #11: Hospital outcomes and blinding
    - 1 time per 150 participants

These are datasets generated through the process of the research and do not reflect data drawn from existing datasets already available through NIH Data Repositories.

- **Scientific data that will be preserved and shared, and the rationale for doing so.** Data to be shared consists of the datasets described above: Data Type (A), which will be shared on the Cancer Data Service (CDS) repository which hosts controlled and open access data. Access to controlled access data on CDS is through the NCI Data Access Committee approved database of Genotypes and Phenotypes (dbGaP) compiled whitelists and will be data of sufficient quality to validate and replicate research findings as described in specific aims.

  Per MD Anderson's NIH Compliant Data Management and Sharing Process, each dataset identified above will be reviewed to comply with the NIH Data Sharing Policy while also satisfying privacy, encumbrances, intellectual property constraints, and the identification of an appropriate data repository.

- **Metadata, other relevant data, and associated documentation.** MD Anderson maintains a robust data management framework to facilitate metadata capture. This will be drawn upon to assure the shared scientific data is appropriately documented while addressing privacy, encumbrances, and intellectual property constraints. In addition, the required technical metadata such as software versions, device models, operating procedures and associated protocol documentation that will facilitate interpretation of the scientific data are captured, documented and maintained with necessary updates within the institutional data and model governance processes. Additional clinically relevant metadata including demographic, diagnosis, and treatment level data are also captured in the institutional data and model governance processes to enable findability and appropriate access to the data for the purposes of scientific reproducibility and reviewed to comply with the NIH Data Sharing Policy.

11.2 Related Tools, Software and/or Code

To facilitate open science, MD Anderson contributes to multiple open-source tools/code. The institutional data and model governance teams oversee an institutional Github repository, dockerized models within XNAT (enterprise installation but available as open-source) as well as common analytic pipelines supported within our data management system, including statistical analytics tools. These facilitate datasets being collected and managed with available codes/platforms.

11.3 Standards

Under the guidance of our institutional policies, MD Anderson maintains robust institutional data and model governance processes for maturing and maintaining mappings to various common data standards (i.e., DICOM for imaging data, AJCC for staging, CTCAE for toxicity, mCODE for clinical

and demographic data), where available. With this institutional approach, scientific data provided will be in alignment with various common data standards, as requested.

11.4 Data Preservation, Access, and Associated Timelines

- **Repository where scientific data and metadata will be archived.** Data to be shared consists of the datasets as described in Section 11.1: Data Type (A). Data repository for these datasets generated from research will be deposited with the CDS, a data repository under the Cancer Research Data Commons infrastructure.

  Per MD Anderson's NIH Compliant Data Management and Sharing Process, each dataset identified above will be reviewed to comply with the NIH Data Sharing Policy while also satisfying privacy, encumbrances, intellectual property constraints, and the identification of an appropriate data repository. Under such process, MD Anderson may identify a more appropriate data repository at the time of release and such information will be provided in the progress report or final report as applicable.

- **How scientific data will be findable and identifiable.** Per MD Anderson's NIH Compliant Data Management and Sharing Process, data will be findable for the research community through appropriate repositories as indicated herein. Summarized data will be made available through peer-reviewed manuscripts, figures, and supplemental materials. At the time of publication, a snapshot of the identified scientific data and necessary metadata will be generated and assigned a digital object identifier (DOI). This data DOI will be referenced in the publication to enable the research community to easily access the data reported in the publication.
- **When and how long the scientific data will be made available.** Per MD Anderson's NIH Compliant Data Management and Sharing Process, the data will be made available to the research community when the award ends. Scientific data made available through the above-listed repositories will remain available as long as the repository remains operational.

11.5 Access, Distribution, or Reuse Considerations

- **Factors affecting subsequent access, distribution, or reuse of scientific data.** Per MD Anderson's NIH Compliant Data Management and Sharing Process, scientific data consisting of human subjects' data will be available for subsequent access, distribution or reuse of such scientific data through the use of controlled access data repositories, where access, distribution or reuse will be limited to comply with Institutional Review Board (IRB)-approved protocols and subjects' informed consent or IRB-approved consent and authorization waiver. Additionally, subsequent access, distribution, or reuse of MD Anderson patient data may be limited because even when safe harbor deidentification standards are met, the patients may still be identifiable based on the uniqueness of the condition or treatments.
- **Whether access to scientific data will be controlled.** Per MD Anderson's NIH Compliant Data Management and Sharing Process, each dataset will undergo institutional review to ensure appropriate controls are in place prior to granting access. For example, raw sequencing data from DNA or RNA of patient germline or tumor samples is considered patient identifiable information and thus will only be released through databases (such as dbGaP) that require the users to follow the principles outlined under the NIH Genomic Data User Code of Conduct. Alternatively, the data generated within this grant contains information that is derived from an IRB-approved clinical trial or based on informed written consent.
- **Protections for privacy, rights, and confidentiality of human research participants.** Per MD Anderson's NIH Compliant Data Management and Sharing Process, scientific data derived from humans will be protected through Certificates of Confidentiality that are automatically issued for NIH-funded human subjects research. In addition, scientific data

derived from humans will be maintained in MD Anderson's HIPAA-compliant data environments. Protective measures for maintaining the security and controlled access to such data housed in these repositories are employed.

11.6 Data Collection and Management Responsibilities

Data Capture: Data will be entered in the MD Anderson institutionally approved database(s). All eligibility criteria must be satisfied prior to treatment initiation.

All data collected will be used only for research purposes. Identifiers (name, medical record number, date of birth, and date of discharge) may be collected. Names and medical record numbers will be replaced by study numbers and dates of birth and death will be replaced by time intervals in the analytic files. Patient identifiers will be confidentially collected and securely maintained on a password-protected server located behind the institutional firewall. Access to identifiers will follow IRB and MD Anderson information security rules and regulations. The master database file will be accessible only to the Principal Investigator, approved co-investigators, and research staff designated on the delegation of authority log.

Accuracy of Data Collection: The MD Anderson Principal Investigator will be the final arbiter of response and toxicity, should a difference of opinion exist.

11.7 Oversight of Data Management and Sharing

Per MD Anderson's NIH Compliant Data Management and Sharing Process, the PI on this project will be responsible for overseeing coordination with the institutional process on all aspects of data management and sharing, including collecting, analyzing, and describing the data, as well as verifying the upload of the data to the repositories listed above. The PI's data manager or other delegate for this project, will be responsible for working with the institutional teams for all data management and sharing, including monitoring adherence to this plan and the NIH Data Management and Sharing policy.

11.8 Incidental/Secondary Findings Disclosure Procedure

Not applicable. No biospecimens, data, or records will be collected to determine any germline and/or somatic mutations.

12 STATEMENT OF COMPLIANCE

NIH-funded investigators and trial site staff who are responsible for the conduct, management, or oversight of NIH-funded trials have completed Human Subjects Protection Training.

The protocol, informed consent form(s), recruitment materials, and all participant materials will be submitted to the IRB for review and approval. Approval of both the protocol and the consent form(s) must be obtained before any participant is consented. Any amendment to the protocol will require review and approval by the IRB before the changes are implemented to the study. All changes to the consent form(s) will be IRB approved; a determination will be made regarding whether a new consent needs to be obtained from participants who provided consent, using a previously approved consent form.

13 REFERENCES

1. Parshall MB, Schwartzstein RM, Adams L, et al. An official American Thoracic Society statement: update on the mechanisms, assessment, and management of dyspnea. *American journal of respiratory and critical care medicine.* 2012;185(4):435-452.
2. Solano JP, Gomes B, Higginson IJ. A comparison of symptom prevalence in far advanced cancer, AIDS, heart disease, chronic obstructive pulmonary disease and renal disease. *J Pain Symptom Manage.* 2006;31(1):58-69.
3. Hui D, Dos Santos R, Chisholm G, Bruera E. Symptom Expression in the Last 7 Days of Life among Cancer Patients Admitted to Acute Palliative Care Units. *J Pain Symptom Manage.* 2015;50(4):488-494
4. Tishelman C, Petersson LM, Degner LF, Sprangers MA. Symptom prevalence, intensity, and distress in patients with inoperable lung cancer in relation to time of death. *Journal of Clinical Oncology.* 2007;25(34):5381-5389.
5. Reddy SK, Parsons HA, Elsayem A, Palmer JL, Bruera E. Characteristics and correlates of dyspnea in patients with advanced cancer. *J Palliat Med.* 2009;12(1):29-36.
6. Maltoni M, Caraceni A, Brunelli C, et al. Prognostic factors in advanced cancer patients: evidence-based clinical recommendations--a study by the Steering Committee of the European Association for Palliative Care. *Journal of Clinical Oncology.* 2005;23(25):6240-6248.
7. Cuervo Pinna MA, Mota Vargas R, Redondo Moralo MJ, Sanchez Correas MA, Pera Blanco G. Dyspnea--a bad prognosis symptom at the end of life. *The American journal of hospice & palliative care.* 2009;26(2):89-97.
8. Caterino JM, Adler D, Durham DD, et al. Analysis of Diagnoses, Symptoms, Medications, and Admissions Among Patients With Cancer Presenting to Emergency Departments. *JAMA Netw Open.* 2019;2(3):e190979.
9. Delgado-Guay MO, Kim YJ, Shin SH, et al. Avoidable and unavoidable visits to the emergency department among patients with advanced cancer receiving outpatient palliative care. *J Pain Symptom Manage.* 2015;49(3):497-504.
10. Stevens JP, Dechen T, Schwartzstein R, et al. Prevalence of Dyspnea Among Hospitalized Patients at the Time of Admission. *J Pain Symptom Manage.* 2018;56(1):15-22.e12.
11. Dudgeon DJ, Kristjanson L, Sloan JA, Lertzman M, Clement K. Dyspnea in cancer patients: prevalence and associated factors. *Journal of Pain and Symptom Management.* 2001;21(2):95-102.
12. Seow H, Barbera L, Sutradhar R, et al. Trajectory of performance status and symptom scores for patients with cancer during the last six months of life. *J Clin Oncol.* 2011;29(9):1151-1158.
13. Currow DC, Smith J, Davidson PM, Newton PJ, Agar MR, Abernethy AP. Do the trajectories of dyspnea differ in prevalence and intensity by diagnosis at the end of life? A consecutive cohort study. *Journal of Pain and Symptom Management.* 2010;39(4):680-690.
14. Hui D, Morgado M, Vidal M, et al. Dyspnea in Hospitalized Advanced Cancer Patients: Subjective and Physiologic Correlates. *Journal of Palliative Medicine.* 2013;16(3):274-280.
15. Board IOMNCP. *Improving Palliative Care for Cancer*. Washington, DC: Institute of Medicine; 2001.
16. Buck H, Brody AA, Campbell ML, et al. Hospice and Palliative Nurses Association 2015-2018 Research Agenda. *Journal of Hospice & Palliative Nursing.* 2015;17(2):119–127.
17. Hui D, Bohlke K, Bao T, et al. Management of Dyspnea in Advanced Cancer: ASCO Guideline. *J Clin Oncol.* 2021;39(12):1389-1411.
18. Hui D, Maddocks M, Johnson MJ, et al. Management of breathlessness in patients with cancer: ESMO Clinical Practice Guidelines. *ESMO Open.* 2020;5(6).
19. Mahler DA. Understanding mechanisms and documenting plausibility of palliative interventions for dyspnea. *Current Opinion in Supportive and Palliative Care.* 2011;5(2):71-76.
20. Spathis A, Booth S, Moffat C, et al. The Breathing, Thinking, Functioning clinical model: a proposal to facilitate evidence-based breathlessness management in chronic respiratory disease. *NPJ primary care respiratory medicine.* 2017;27(1):27.
21. Lovell N, Etkind SN, Bajwah S, Maddocks M, Higginson IJ. Control and Context Are Central for People With Advanced Illness Experiencing Breathlessness: A Systematic Review and Thematic Synthesis. *J Pain Symptom Manage.* 2019;57(1):140-155.e142.

22. Hui D, Hernandez F, Urbauer D, et al. High Flow Oxygen and High Flow Air for Dyspnea in Hospitalized Patients with Cancer: A Pilot Crossover Randomized Clinical Trial. *Oncologist.* 2021;26(5):e883–892.

23. Hui D, Morgado M, Chisholm G, et al. High-flow oxygen and bilevel positive airway pressure for persistent dyspnea in patients with advanced cancer: a phase II randomized trial. *J Pain Symptom Manage.* 2013;46(4):463-473.

24. Benditt JO. Adverse effects of low-flow oxygen therapy. *Respir Care.* 2000;45(1):54-61; discussion 61-54.

25. Cornet AD, Kooter AJ, Peters MJ, Smulders YM. The potential harm of oxygen therapy in medical emergencies. *Crit Care.* 2013;17(2):313.

26. Nishimura M. High-flow nasal cannula oxygen therapy in adults. *J Intensive Care.* 2015;3(1):15.

27. Code of Federal Regulations Title 21. 2018; https://www.accessdata.fda.gov/scripts/cdrh/cfdocs/cfcfr/cfrsearch.cfm?fr=868.5454. Accessed November 16, 2023, 2023.

28. Hui D, Park M, Shamieh O, et al. Personalized symptom goals and response in patients with advanced cancer. *Cancer.* 2016;122(11):1774-1781.

29. Mercadante S, Adile C, Aielli F, et al. Personalized Goal for Dyspnea and Clinical Response in Advanced Cancer Patients. *J Pain Symptom Manage.* 2019;57(1):79-85.

30. Carvajal A, Centeno C, Watson R, Bruera E. A comprehensive study of psychometric properties of the Edmonton Symptom Assessment System (ESAS) in Spanish advanced cancer patients. *Eur J Cancer.* 2011;47(12):1863-1872.

31. Hui D, Bruera E. The Edmonton Symptom Assessment System 25 Years Later: Past, Present, and Future Developments. *J Pain Symptom Manage.* 2017;53(3):630-643.

32. EuroQol. EQ-5D-5L: Available modes of administration. 2017; https://euroqol.org/eq-5d-instruments/eq-5d-5l-available-modes-of-administration/. Accessed October 3, 2017, 2017.

33. Hernandez G, Garin O, Pardo Y, et al. Validity of the EQ-5D-5L and reference norms for the Spanish population. *Qual Life Res.* 2018;27(9):2337-2348.

34. Lin FJ, Pickard AS, Krishnan JA, et al. Measuring health-related quality of life in chronic obstructive pulmonary disease: properties of the EQ-5D-5L and PROMIS-43 short form. *BMC Med Res Methodol.* 2014;14:78.

35. Busner J, Targum SD. The clinical global impressions scale: applying a research tool in clinical practice. *Psychiatry (Edgmont (Pa. : Township)).* 2007;4(7):28-37.

36. Redelmeier DA, Guyatt GH, Goldstein RS. Assessing the minimal important difference in symptoms: a comparison of two techniques. *Journal of clinical epidemiology.* 1996;49(11):1215-1219.

37. Guyatt GH, Feeny DH, Patrick DL. Measuring health-related quality of life. *Annals of Internal Medicine.* 1993;118(8):622-629.

38. Hui D, Shamieh O, Paiva C, et al. Minimal Clinically Important Differences in the Edmonton Symptom Assessment Scale in Cancer Patients: A Prospective Study. *Cancer.* 2015;121(17):3027-3035.

39. Hui D, Puac V, Shelal Z, et al. Effect of dexamethasone on dyspnoea in patients with cancer (ABCD): a parallel-group, double-blind, randomised, controlled trial. *Lancet Oncol.* 2022;23(10):1321-1331.
