# Supplementary material for: Structured Personalized Oxygen and Supportive Therapies for Dyspnea in Oncology (SPOT-ON): A personalized randomized clinical trial protocol
Source: PLoS One. 2025 Dec 2;20(12):e0336691. doi: 10.1371/journal.pone.0336691 (PMC12671826; doi:10.1371/journal.pone.0336691)
Supplement: S3 File — (PDF) [file pone.0336691.s003.pdf]

Informed Consent/Authorization for Participation in Research

**Title of Research Study:**	Structured Personalized Oxygen and Supportive Therapies for Dyspnea in Oncology (SPOT-ON) Approach for Dyspnea Treatment in Cancer Patients: A Randomized Clinical Trial
**Subtitle:**	SPOT-ON
**Study Number:**	2023-0933
**Principal Investigator:**	David Hui, MD

__________________________ Participant's Name

__________________________ Medical Record Number

Key Information

The following is a short summary of this study to help you decide whether or not to be a part of this study. More detailed information is listed later on in this form.

Why am I being invited to take part in a research study?

You are invited to take part in a research study because you have advanced cancer and are experiencing difficulty breathing (called dyspnea).

What should I know about a research study?

- Someone will explain this research study to you.
- Whether or not you take part is up to you.
- You can choose not to take part.
- You can agree to take part and later change your mind.
- Your decision will not be held against you.
- You can ask all the questions you want before you decide.

Why is this research being done?

Currently, there is still much debate about which of the treatments for shortness of breath are most effective for individual patients. In this study, patients will try multiple therapies to identify the best combination for relieving their shortness of breath.

The goal of this clinical research study is to learn about the effect of Structured Personalized Oxygen and Supportive Therapies for Dyspnea in Oncology (SPOT-ON) treatment on the severity of shortness of breath in patients with cancer.

This is an investigational study. SPOT-ON is an investigational approach for deciding the best shortness of breath treatment for cancer patients. Study therapy will be delivered using standard, FDA-approved devices.

The study doctor can answer questions about the SPOT-ON treatment.

How long will the research last and what will I need to do?

You will receive SPOT-ON treatment for 72 hours (3 days). About 30 days after your treatment ends, the study staff will check on how you are doing.

You will be asked to work with the research team to complete the treatment and answer questionnaires.

More detailed information about the study procedures can be found under ***"What happens if I agree to be in this research?"***

Is there any way being in this study could be bad for me?

Before choosing to take part in this study, you should discuss with the study team any concerns you may have, including side effects, potential expenses, and time commitment.

More detailed information about the risks of this study can be found under ***"Is there any way being in this study could be bad for me? (Detailed Risks)"***

Will being in this study help me in any way?

Taking part in this study can help provide relief for your shortness of breath. Future patients may benefit from what is learned. However, it cannot be promised that there will be any benefits to you or others from your taking part in this research.

What happens if I do not want to be in this research?

Participation in research is completely voluntary. You can decide to participate, not participate, or stop participation at any time without penalty or loss of your regular benefits.

Instead of taking part in this study, you may choose to receive standard care for shortness of breath outside of this study. You may choose to receive other investigational care, if available. These alternatives have risks and benefits that may be the same or different than those in this research study. The study doctor can discuss these alternatives with you, including their possible risks and benefits.

In all cases, you will receive appropriate medical care.

Detailed Information

The following is more detailed information about this study in addition to the information listed above.

Who can I talk to if I have questions or concerns?

If you have questions, concerns, or complaints, or think the research has hurt you, talk to the research team at 713-563-7637.

This research has been reviewed and approved by the MD Anderson Institutional Review Board (IRB – an ethics committee that reviews research studies). You may talk to them at 713-792-6477 or IRB_Help@mdanderson.org if:

- Your questions, concerns, or complaints are not being answered by the research team.
- You cannot reach the research team.
- You want to talk to someone besides the research team.
- You have questions about your rights as a research participant.
- You want to get information or provide input about this research.

How many people will be in this study?

It is expected about 150 people at MD Anderson will be enrolled in this research study.

What happens if I agree to be in this research?

Study Groups
If you agree to take part in this study, you will be randomly assigned (as in the flip of a coin) to 1 of 2 study groups (SPOT-ON or SPOT-ON Waitlist) to determine when you will start SPOT-ON treatment for your shortness of breath. You will have an equal

chance (50/50) of being assigned to either group. Randomization will be completed by a computer using the secure Clinical Trial Conduct website.

You will start the SPOT-ON treatment within 3 days after you are enrolled in the study; however, the exact timing of when this treatment will start will be based on the study group you are assigned to. To minimize bias in the study, the exact timing you will receive SPOT-ON treatment will not be disclosed to you or the research coordinator doing the study assessments.

Study Treatment
During the study treatment, you will work with the healthcare team to find the best ways to reduce your shortness of breath by finding what combination of treatments work for you. You will receive education about shortness of breath by the research staff in addition to information about standard treatments or medications for your shortness of breath that would be offered by your medical team.

Regardless of which study group you are assigned to, you will start treatment within 3 days of enrollment, which may include: receiving information on breathing techniques, relaxation techniques, posture techniques, and distraction techniques, and/or trying different oxygen-based therapies, such as high-flow nasal cannula, low-flow supplemental oxygen, and non-invasive ventilation with a respiratory therapist.

High-flow nasal cannula delivers warm, humidified oxygen through a heated tube through the nostrils. Low-flow supplemental oxygen is not warmed and is delivered at a slower rate than high-flow. Non-invasive ventilation involves the delivery of oxygen into the lungs through a face or nasal mask under positive pressure, without the need for intubation.

All oxygen-based therapies are currently used in clinical care. High-flow nasal cannula and non-invasive ventilation will be delivered using an FDA-approved Hamilton C1 ventilator with commercially available accessories, including nasal masks, facial masks, and nasal cannula. This device is currently used throughout MD Anderson for routine clinical care.

If you choose to sample oxygen-based therapies, you will have the opportunity to try each of the 3 treatments for 5–10 minutes. Afterwards, you will have a say in which one(s), if any, you would like to continue using for the management of your shortness of breath over the next 72 hours.

You are not required to try any of these oxygen-based therapies if you do not want to, and you can still participate in the study if you choose not to try an oxygen-based therapy.

You will have completed the study treatment after you have completed 72 hours of SPOT-ON treatment or if you ask to be removed from the study.

Study Questionnaires
Before starting the treatment, the study staff will collect information about your demographics (such as your sex, ethnicity, and race), your cancer diagnosis, other diseases or conditions you may have, measurements related to your breathing (such as blood oxygen level and the amount of air you can inhale), medications you are receiving, other symptoms you may have, and your respiratory care goal. You will answer questionnaires about the intensity and unpleasantness of your shortness of breath, symptoms you are experiencing, and your quality of life. They will take less than 10 minutes total to complete.

Your shortness of breath and other symptoms will be assessed by the research coordinators through questionnaires that will be done over the telephone. At 24, 48, and 72 hours, you will answer the same questionnaires you answered before starting this clinical trial, as well as questionnaires about changes in your ability to breathe and your experience in the study. They will take less than 10 minutes total to complete. The study team will also ask you if you have experienced any side effects.

Follow-Up
About 30 days after your treatment ends, the study staff will call you to check on how you are doing and ask if you have experienced any side effects. The call will take about 5 minutes.

If you stop taking part in this study because of intolerable side effect(s), you will be followed until the side effect(s) get better or become stable.

What are my responsibilities if I take part in this research?

If you take part in this research, you will be responsible for the following:
- Tell the study team about any symptoms or side effects you have.
- Follow study directions.

What happens if I say yes, but I change my mind later?

You can leave the research at any time; it will not be held against you. You may withdraw from participation in this study without any penalty or loss of benefits. If you withdraw from this study, you can still choose to be treated at MD Anderson.

If you decide you want to stop taking part in the study, it is recommended for your safety that you first talk to your doctor who can help you safely stop study treatment. It may be dangerous to suddenly stop study treatment. The study doctor will also decide if you need to have any visits or tests to check on your health.

If you stop participating in the research study, already collected data may not be removed from the study database. You may be asked whether the study doctor can

collect data from your routine medical care. If you agree, this data will be handled the same as research data.

Is there any way being in this study could be bad for me? (Detailed Risks)

While on this study, you are at risk for side effects. These side effects will vary from person to person. The more commonly occurring side effects are listed in this form, as are rare but serious side effects. You should discuss these with the study doctor. You may also want to ask about uncommon side effects that have been observed in small numbers of patients but are not listed in this form. Many side effects go away shortly after the procedure, but in some cases side effects may be serious, long-lasting, or permanent, and may even result in hospitalization and/or death.

Tell the study staff about any side effects you may have, even if you do not think they are related to the procedure.

Supplemental Oxygen Use Side Effects (high-flow nasal canula, non-invasive ventilation, and low-flow supplemental oxygen)

High-flow nasal canula, non-invasive ventilation, and low-flow supplemental oxygen involve delivering extra oxygen to you. The use of supplemental oxygen may cause:

• irritated or dry mouth or throat<br>• eye irritation (possible dry eyes or painful red eyes)	• difficulty breathing<br>• irritated or dry nose	• oxygen toxicity (possible damage to lungs, eyes, and nervous system, especially with prolonged exposure to high levels of oxygen)

There is an increased risk of fire, especially if the patient is smoking or if oxygen leaks from the delivery device and makes contact with an ignition source.

High-flow Nasal Canula Side Effects

High-flow nasal canula may cause nasal bleeding and an increased risk of infection, such as pneumonia. This infection may occur anywhere. It may become life-threatening. Symptoms of infection may include fever, pain, redness, and difficulty breathing.

Non-invasive Ventilation Side Effects

Non-invasive ventilation may cause the following side effects:

• discomfort from mask or skin irritation behind the ears or under the nose<br>• difficulty tolerating the pressure support, such as air hunger, chest pain, or abdominal swelling	• increased work of breathing or airway resistance, which may lead to failure of non-invasive ventilation and need for invasive ventilation	• worsening of the underlying respiratory or cardiac condition, such as increased oxygen requirement or fluid in the lung<br>• infection

Non-invasive ventilation may cause an increased risk of infection, such as pneumonia or sinusitis. This infection may occur anywhere. It may become life-threatening. Symptoms of infection may include fever, pain, redness, and difficulty breathing.

Other Risks

Questionnaires may contain questions that are sensitive in nature. You may refuse to answer any question that makes you feel uncomfortable. If you have concerns about completing the questionnaire, you are encouraged to contact your doctor or the study chair.

Although every effort will be made to keep study data safe, there is a chance that your personal health information could be lost or stolen, which may result in a **loss of confidentiality**. All study data will be stored in password-protected computers and/or locked file cabinets and will continue to be stored securely after the study.

In addition to these risks, this research may hurt you in ways that are unknown. These may be a minor inconvenience or may be so severe as to cause death.

You will be told about any new information that may affect your health, welfare, or choice to stay in the research.

Will it cost anything to be in this study? Will I be paid to be in this study?

The SPOT-ON treatments offered by the respiratory therapist will be provided at no cost to you.

You and/or your insurance provider will not have to pay for certain research exams and procedures done that are covered by the study.

You and/or your insurance provider will be responsible for the costs of routine clinical services (such as diagnostic/therapeutic procedures, drugs, devices, laboratory assays, and other services that would ordinarily be ordered for medical care, regardless of whether or not you are participating in a study). There may be extra costs that are not covered by your medical plan that you will have to pay yourself.

Taking part in this study may result in added costs to you (such as transportation, parking, meals, or unpaid leave from work). You may have to pay for medication prescribed to treat or prevent side effects, and you may have to visit the clinic/hospital more often than if you were not participating in this study.

If you have insurance, talk to your insurance provider, and make sure that you understand what your insurance pays for and what it does not pay for if you take part in this study. Also, find out if you need approval from your plan before you can take part in the study.

You may ask that a financial counselor be made available to you to talk about the costs of this study.

As compensation for your time and effort, you will receive a $50 gift card for completing the study.

What happens to the information collected for the research?

Efforts will be made to limit the use and disclosure of your personal information, including research study and medical records, to people who need to review this information. Complete secrecy cannot be promised. Organizations that may inspect and copy your information include the IRB and other representatives of this organization.

A participant study number will be assigned to you once you have been enrolled in the study. This participant study number will be used to identify your data in the study report and when reporting any data from the study.

Any personal information that could identify you will be removed or changed before data are shared with other researchers or results are made public.

The results of this research may be published in scientific journals or presented at medical meetings. However, your identity will not be disclosed. Your name and other identifying information will be kept confidential.

This research is covered by a Certificate of Confidentiality from the National Institutes of Health. This means that the researchers cannot release or use information, documents, or samples that may identify you in any action or suit unless you say it is okay. They also cannot provide them as evidence unless you have agreed. This protection includes federal, state, or local civil, criminal, administrative, legislative, or other proceedings. An example would be a court subpoena.

There are some important things that you need to know. The Certificate DOES NOT stop reporting that federal, state, or local laws require. Some examples are laws that require reporting of child or elder abuse, some communicable diseases, and threats to

harm yourself or others. The Certificate CANNOT BE USED to stop a sponsoring United States federal or state government agency from checking records or evaluating programs. The Certificate DOES NOT stop disclosures required by the federal Food and Drug Administration (FDA). The Certificate also DOES NOT prevent your information from being used for other research if allowed by federal regulations.

Researchers may release information about you when you say it is okay. For example, you may give them permission to release information to insurers, medical providers or any other persons not connected with the research. The Certificate of Confidentiality does not stop you from willingly releasing information about your involvement in this research. It also does not prevent you from having access to your own information.

The sponsor, monitors, auditors, the IRB, and the Food and Drug Administration will be granted direct access to your medical records to conduct and oversee the research. By signing this document, you are authorizing this access.

Federal law provides additional protections of your medical records and related health information. These are described below.

Will my data or samples be used for future research?

Your personal information and/or samples are being collected as part of this study. These data and/or samples may be used by researchers at MD Anderson and the National Institutes of Health, or shared with other researchers and/or institutions for use in future research.

In some cases, all of your identifying information may not be removed before your data or research samples are used for future research. If future research is performed at MD Anderson, the researchers must get approval from the MD Anderson IRB before your data and/or research samples can be used. At that time, the IRB will decide whether or not further permission from you is required. If this research is not performed at MD Anderson, MD Anderson will not have oversight of any data and/or samples.

If identifiers are removed from your identifiable private information or identifiable samples that are collected during this research, that information or those samples could be used for future research studies or shared with another researcher for future research studies without your additional informed consent.

Can I be removed from the research study without my permission?

The person in charge of the research study or the sponsor can remove you from the research study without your approval. Possible reasons for removal include if the disease gets worse, if intolerable side effects occur, if you are unable to follow study directions, or if the study is stopped.

What happens if I get hurt from being in this study?

If you get sick or hurt and it is related to your participation in this study, you will be given care at MD Anderson (if you are at the clinic when you are sick or hurt). If you get hurt or sick and you are not at the clinic (for example, you are at home or at another doctor's office):

- call your personal doctor right away (or in an emergency, call 911)
- tell your personal doctor or ER staff that you are in this study (try to give them a copy of this consent form or show them your participant card)
- call the study doctor (Dr. David Hui, at 832-421-4450) or 713-792-2121 (24 hours)

You will not be reimbursed for expenses or compensated financially by MD Anderson or the National Institutes of Health for this injury. Costs of treatment received because you were hurt or sick will be billed to you or your insurance company. No other form of payment is available.

You may also contact the MD Anderson IRB at 713-792-6477 with questions about study-related injuries. By signing this consent form, you are not giving up any of your legal rights.

What else do I need to know?

This research is being funded by the National Institutes of Health

MD Anderson may benefit from your participation and/or what is learned in this study.

Your information (both identifiable and de-identified) may be used to create products or to deliver services, including some that may be sold and/or make money for others. If this happens, there are no plans to tell you, or to pay you, or to give any compensation to you or your family.

Authorization for Use and Disclosure of Protected Health Information (PHI):

A. During the course of this study, MD Anderson will be collecting and using your PHI, including identifying information, information from your medical record, and study results. For legal, ethical, research, and safety-related reasons, your doctor and the research team may share your PHI with:
    - Federal agencies that require reporting of clinical study data (such as the FDA, National Cancer Institute [NCI], and OHRP)
    - The IRB and officials of MD Anderson
    - National Institutes of Health, who is a sponsor or supporter of this study, and/or any future sponsors/supporters of the study, and/or licensees of the study technology

- Study monitors and auditors who verify the accuracy of the information
- Individuals who put all the study information together in report form

Study sponsors and/or supporters receive limited amounts of PHI. They may also view additional PHI in study records during the monitoring process. MD Anderson's contracts require sponsors/supporters to protect this information and limit how they may use it.

B. Signing this consent and authorization form is optional but you cannot take part in this study or receive study-related treatment if you do not agree and sign.

C. MD Anderson will do its best to protect the privacy of your records, but it is possible that once information is shared with people listed on this form, it may be released to others. If this happens, your information may no longer be protected by federal law.

D. The permission to use your PHI will continue indefinitely unless you withdraw your authorization in writing. Instructions on how to do this can be found in the MD Anderson Notice of Privacy Practices (NPP) or you may contact the Chief Privacy Officer at 713-745-6636. If you withdraw your authorization, you will be removed from the study and the data collected about you up to that point can be used and included in data analysis. However, no further information about you will be collected.

E. A description of this clinical trial will be available on http://www.ClinicalTrials.gov, as required by U.S. Law. This Web site will not include information that can identify you. At most, the Web site will include a summary of the results. You can search this Web site at any time.

CONSENT/AUTHORIZATION

I understand the information in this consent form. I have had a chance to read the consent form for this study, or have had it read to me. I have had a chance to think about it, ask questions, and talk about it with others as needed. I give the study chair permission to enroll me on this study. By signing this consent form, I am not giving up any of my legal rights. I will be given a signed copy of this consent document.

_____________________________________ _______
SIGNATURE OF PARTICIPANT DATE

____
PRINTED NAME OF PARTICIPANT

WITNESS TO CONSENT

I was present during the explanation of the research to be performed under this protocol.

____ _________
SIGNATURE OF WITNESS TO THE VERBAL CONSENT DATE
PRESENTATION (OTHER THAN PHYSICIAN OR STUDY CHAIR)
A witness signature is only required for non-English speakers utilizing the short form consent process (VTPS) and patients who are illiterate.

____
PRINTED NAME OF WITNESS TO THE VERBAL CONSENT

PERSON OBTAINING CONSENT

I have discussed this research study with the participant and/or his or her authorized representative, using language that is understandable and appropriate. I believe that I have fully informed this participant of the nature of this study and its possible benefits and risks and that the participant understood this explanation.

____ _________
PERSON OBTAINING CONSENT DATE

____
PRINTED NAME OF PERSON OBTAINING CONSENT

TRANSLATOR

I have translated the above informed consent as written (without additions or subtractions) into____________________________and assisted the people
(Name of Language)
obtaining and providing consent by translating all questions and responses during the consent process for this participant.

NAME OF TRANSLATOR	SIGNATURE OF TRANSLATOR	DATE

☐ Please check here if the translator was a member of the research team. (If checked, a witness, other than the translator, must sign the witness line.)

Consentimiento informado/autorización para participar en una investigación

**Título del estudio de investigación:**	Enfoque estructurado personalizado de oxígeno y terapias de apoyo para la disnea en oncología (SPOT-ON) para el tratamiento de la disnea en pacientes con cáncer: ensayo clínico aleatorizado
**Subtítulo:**	SPOT-ON
**Número del estudio:**	2023-0933
**Investigador principal:**	David Hui, MD

____________________________________ __________________________
Nombre del/de la participante Número de expediente médico

Información clave

Este es un breve resumen del estudio para ayudarlo/a a decidir si desea participar. Más adelante en este formulario se presenta información más detallada.

¿Por qué me invitan a participar en un estudio de investigación?

Lo/la invitan a participar en un estudio de investigación porque padece un cáncer avanzado y experimenta dificultad para respirar (denominada "disnea").

¿Qué debo saber sobre un estudio de investigación?

- Una persona le explicará este estudio de investigación.
- Participar o no es su decisión.
- Puede elegir no participar.
- También puede elegir participar y cambiar de opinión más adelante.
- Su decisión no se usará en su contra.
- Puede hacer todas las preguntas que desee antes de tomar su decisión.

¿Por qué se hace esta investigación?

Actualmente sigue habiendo mucho debate sobre cuál de los tratamientos para la falta de aire es más eficaz para cada paciente. En este estudio, los pacientes probarán múltiples terapias con el fin de identificar la mejor combinación para aliviar su falta de aire.

El objetivo de este estudio de investigación clínica es conocer el efecto del tratamiento con oxígeno estructurado personalizado y las terapias de apoyo para la disnea en oncología (SPOT-ON por sus siglas en inglés) en la gravedad de la falta de aire en pacientes con cáncer.

Este es un estudio de investigación. SPOT-ON es un enfoque en investigación para decidir el mejor tratamiento de la falta de aire en pacientes con cáncer. La terapia del estudio se administrará utilizando dispositivos estándar aprobados por la FDA.

El médico del estudio podrá responder a sus preguntas sobre el tratamiento SPOT-ON.

¿Cuánto durará la investigación y qué tendré que hacer?

Recibirá el tratamiento SPOT-ON durante 72 horas (3 días). Unos 30 días después de finalizar el tratamiento, el personal del estudio comprobará cómo se encuentra.

Le pedirán que colabore con el equipo de investigación para completar el tratamiento y responder a los cuestionarios.

Encontrará información más detallada sobre los procedimientos del estudio en la sección ***“¿Qué ocurre si acepto participar en esta investigación?”***.

¿Participar en este estudio puede perjudicarme de alguna manera?

Antes de decidir participar en este estudio, hable con el equipo del estudio sobre las dudas que tenga, así como sobre los efectos secundarios, los posibles gastos y el compromiso de tiempo.

Encontrará información más detallada sobre los riesgos del estudio en el apartado ***“¿Participar en este estudio puede perjudicarme de alguna manera? (Riesgos detallados)”***.

¿Participar en este estudio me ayudará de alguna manera?

Participar en este estudio puede ayudar a aliviar su falta de aire. Los futuros pacientes podrían beneficiarse de lo que se aprenda. Sin embargo, no se puede prometer que su participación en esta investigación suponga un beneficio para usted ni para otras personas.

¿Qué ocurre si no quiero participar en esta investigación?

La participación en una investigación es totalmente voluntaria. Puede elegir participar, no participar o dejar de participar en cualquier momento sin recibir sanciones ni perder ninguno de sus beneficios habituales.

En lugar de participar en este estudio, puede optar por recibir atención estándar para la falta de aire fuera de este estudio. Puede optar por recibir otros cuidados en investigación, si están disponibles. Estas alternativas tienen riesgos y beneficios que pueden ser iguales o diferentes a los de este estudio de investigación. El médico del estudio puede hablar sobre estas alternativas con usted, incluidos sus posibles riesgos y beneficios.

En todos los casos recibirá la atención médica apropiada.

Información detallada

A continuación, encontrará información más detallada sobre este estudio, además de la información indicada anteriormente.

¿Con quién puedo hablar si tengo preguntas o inquietudes?

Si tiene preguntas, preocupaciones o quejas, o cree que la investigación lo/la ha perjudicado, hable con el equipo de investigación llamando al 713-563-7637.

Esta investigación ha sido revisada y aprobada por la Institutional Review Board (Junta de Revisión Institucional, o IRB, por sus siglas en inglés; un comité de ética que revisa los estudios de investigación) de MD Anderson. Puede hablar con ellos llamando al 713-792-6477 o escribiendo a IRB_Help@mdanderson.org si ocurre lo siguiente:

- El equipo de investigación no responde a sus preguntas, inquietudes o quejas.
- No puede comunicarse con el equipo de investigación.
- Quiere hablar con alguien que no sea parte del equipo de investigación.
- Tiene preguntas sobre sus derechos como participante en la investigación.
- Desea información o dar su opinión sobre esta investigación.

¿Cuántas personas participarán en este estudio?

Se espera que alrededor de 150 personas en MD Anderson se inscriban en este estudio de investigación.

¿Qué ocurre si acepto participar en esta investigación?

Tratamiento del estudio

Durante el tratamiento SPOT-ON de tres días, trabajará con el equipo de atención de la salud para encontrar la mejor manera de reducir su falta de aire encontrando la combinación

de tratamientos que funciona para usted. Esto incluirá probar diferentes terapias basadas en el oxígeno, como la cánula nasal, el oxígeno suplementario y la ventilación no invasiva con un terapeuta respiratorio. Además del tratamiento SPOT-ON, nuestro personal de investigación le dará cierta educación básica sobre la falta de aire y su equipo asistencial habitual le dará tratamientos estándar para su falta de aire. *Todos los pacientes del estudio comenzarán el tratamiento SPOT-ON para la falta de aire en un plazo de tres días después de su inscripción;* sin embargo, una computadora asignará aleatoriamente el momento exacto en que se iniciará este tratamiento (como al lanzar una moneda al aire).

Cuestionarios del estudio

Antes de iniciar el tratamiento, el personal del estudio recopilará información sobre sus datos demográficos (como su sexo, etnia y raza), su diagnóstico de cáncer, otras enfermedades o afecciones que pueda padecer, mediciones relacionadas con su respiración (como el nivel de oxígeno en sangre y la cantidad de aire que puede inhalar), los medicamentos que está recibiendo, otros síntomas que pueda tener y su objetivo de cuidados respiratorios. Responderá a cuestionarios sobre la intensidad y lo desagradable que le resulta su falta de aire, los síntomas que experimenta y su calidad de vida. Rellenarlos le tomará menos de 10 minutos.

Los coordinadores de la investigación evaluarán su dificultad para respirar y otros síntomas mediante cuestionarios que se realizarán por teléfono. A las 24, 48 y 72 horas, responderá los mismos cuestionarios que contestó antes de empezar este ensayo clínico, así como los cuestionarios sobre los cambios en su capacidad para respirar y su experiencia en el estudio. Rellenarlos le tomará menos de 10 minutos. El equipo del estudio también le preguntará si ha experimentado algún efecto secundario.

Seguimiento
Unos 30 días después de finalizar el tratamiento, el personal del estudio lo/la llamará para comprobar cómo se encuentra y preguntarle si ha experimentado algún efecto secundario. La llamada durará unos 5 minutos.

Si deja de participar en este estudio debido a efectos secundarios intolerables, le harán un seguimiento hasta que los efectos secundarios mejoren o se estabilicen.

¿Cuáles son mis responsabilidades si participo en esta investigación?

Si decide participar en esta investigación, sus responsabilidades serán las siguientes:

- Informar al equipo del estudio sobre cualquier síntoma o efecto secundario que tenga.
- Seguir las instrucciones del estudio.

¿Qué pasa si digo que sí, pero luego cambio de opinión?

Puede abandonar la investigación en cualquier momento, esto no se usará en su contra. Puede retirarse de la participación en este estudio sin ninguna penalización ni pérdida de

beneficios. Aunque se retire de este estudio, podrá elegir recibir tratamiento en MD Anderson.

Si decide que quiere dejar de participar en el estudio, se recomienda por su seguridad que hable primero con su médico, quien puede ayudarlo/a a interrumpir el tratamiento del estudio de forma segura. Puede ser peligroso interrumpir repentinamente el tratamiento del estudio. El médico del estudio también decidirá si necesita someterse a alguna visita o prueba para controlar su salud.

Si deja de participar en el estudio de investigación, los datos ya recogidos no podrán eliminarse de la base de datos del estudio. Es posible que le pregunten si el médico del estudio puede recopilar datos de su atención médica de rutina. Si está de acuerdo, estos datos se manejarán igual que los de la investigación.

¿Participar en este estudio puede perjudicarme de alguna manera? (Riesgos detallados)

Mientras participe en este estudio, usted corre el riesgo de tener efectos secundarios, Estos efectos secundarios variarán de una persona a otra. Los efectos secundarios más comunes se encuentran enumerados en este formulario, al igual que los poco frecuentes, pero graves. Debe hablar sobre ellos con el médico del estudio. También le sugerimos que pregunte acerca de los efectos secundarios poco comunes que se hayan observado en un número escaso de pacientes, pero que no estén enumerados en este formulario. Muchos efectos secundarios desaparecen poco después del procedimiento, pero en algunos casos pueden ser graves, duraderos o permanentes, y pueden incluso requerir hospitalización y/o causar la muerte.

Informe al personal del estudio de cualquier efecto secundario que pueda tener, incluso si considera que no está relacionado con el procedimiento.

<u>Efectos secundarios del uso de oxígeno suplementario (cánula nasal de alto flujo, ventilación no invasiva y oxígeno suplementario de bajo flujo)</u>

La cánula nasal de alto flujo, la ventilación no invasiva y el oxígeno suplementario de bajo flujo consisten en suministrarle oxígeno adicional. El uso de oxígeno suplementario puede provocar lo siguiente:

• irritación o sequedad en la boca o la garganta<br>• irritación de los ojos (posible sequedad de los ojos o enrojecimiento doloroso de los ojos)	• dificultad para respirar<br>• irritación o sequedad en la nariz	• toxicidad del oxígeno (posibles daños en los pulmones, los ojos y el sistema nervioso, sobre todo con una exposición prolongada a altos niveles de oxígeno)

Existe un mayor riesgo de incendio, especialmente si el/la paciente fuma o si el oxígeno se escapa del dispositivo de suministro y entra en contacto con una fuente de ignición.

Efectos secundarios de la cánula nasal de alto flujo

La cánula nasal de alto flujo puede provocar sangrado nasal y un mayor riesgo de infección, como la neumonía. Esta infección puede ocurrir en cualquier parte y poner en peligro la vida. Los síntomas de infección pueden incluir fiebre, dolor, enrojecimiento y dificultad para respirar.

Efectos secundarios de la ventilación no invasiva

La ventilación no invasiva puede provocar los siguientes efectos secundarios:

• molestias por la mascarilla o irritación de la piel detrás de las orejas o debajo de la nariz<br>• dificultad para tolerar el soporte de presión, como hambre de aire, dolor torácico o hinchazón abdominal	• aumento del trabajo respiratorio o de la resistencia de las vías respiratorias, que puede provocar el fracaso de la ventilación no invasiva y la necesidad de ventilación invasiva	• empeoramiento de la afección respiratoria o cardiaca subyacente, como una mayor necesidad de oxígeno o la presencia de líquido en el pulmón<br>• infección

La ventilación no invasiva puede provocar un mayor riesgo de infección, como neumonía o sinusitis. Esta infección puede ocurrir en cualquier parte y poner en peligro la vida. Los síntomas de infección pueden incluir fiebre, dolor, enrojecimiento y dificultad para respirar.

Otros riesgos

Los **cuestionarios** pueden contener preguntas de naturaleza sensible. Usted puede negarse a responder cualquier pregunta que le haga sentir incomodidad. Si tiene alguna pregunta acerca de cómo contestar el cuestionario, le aconsejamos que contacte a su médico o al investigador principal.

Aunque se tomarán todas las medidas posibles para mantener la seguridad de los datos del estudio, existe la posibilidad de que su información de salud personal se pierda o sea robada, lo que puede dar lugar a una **pérdida de confidencialidad**. Todos los datos del estudio se almacenarán en computadoras protegidas por contraseña o armarios cerrados con llave, y permanecerán almacenados de manera segura después del estudio.

Además de estos riesgos, esta investigación puede perjudicarlo/a de formas que aún se desconocen. Esto podría ser desde una inconveniencia leve hasta una consecuencia tan grave que ocasione la muerte.

Le comunicaremos cualquier información nueva que pudiera afectar su salud, su bienestar o su deseo de permanecer en este estudio.

¿Cuál será el costo de participar en este estudio? *¿Me pagarán por participar en este estudio?*

Los tratamientos SPOT-ON ofrecidos por el/la terapeuta respiratorio le serán proporcionados de forma gratuita para usted.

Ni usted ni su compañía de seguros tendrán que pagar por ciertos procedimientos y exámenes de investigación que estén cubiertos por el estudio.

Usted y/o su compañía de seguros serán responsables de los costos de los servicios clínicos de rutina (como procedimientos diagnósticos/terapéuticos, medicamentos, dispositivos, ensayos de laboratorio y otros servicios que normalmente se solicitarían para la atención médica, independientemente de si participa o no en un estudio). Puede haber otros costos que no cubra su plan médico y que deberá pagar usted.

Participar en este estudio puede suponerle costos añadidos (como transporte, estacionamiento, comidas o permisos no remunerados en el trabajo). Es posible que tenga que pagar la medicación que le receten para tratar o prevenir los efectos secundarios y que tenga que acudir a la clínica o al hospital con más frecuencia que si no participara en este estudio.

Si tiene seguro, hable con su compañía de seguros y asegúrese de que entiende lo que paga su seguro y lo que no paga si participa en este estudio. Además, averigüe si necesita la aprobación previa de su plan para poder participar en el estudio.

Usted puede pedir que le pongan a su disposición un/a asesor/a de finanzas para que hable con usted sobre los costos de este estudio.

Como compensación por su tiempo y esfuerzo, recibirá una tarjeta regalo de 50 dólares por completar el estudio.

¿Qué ocurre con la información recopilada para la investigación?

Se hará todo lo posible para limitar el uso y la divulgación de su información personal, incluidos los expedientes médicos y del estudio de investigación, a las personas que necesitan revisar esta información. No podemos garantizar el secreto total. Entre las organizaciones que pueden inspeccionar y copiar su información, se incluyen la IRB y otros representantes de esta organización.

Una vez se haya inscrito en el estudio, le asignarán un número de participante del estudio. Este número de participante del estudio se utilizará para identificar sus datos en el informe del estudio y al comunicar cualquier dato del estudio.

Se eliminará o modificará cualquier información personal que permita su identificación antes de que se compartan los datos con otros investigadores o de que se hagan públicos los resultados.

Los resultados de esta investigación podrán publicarse en revistas científicas o presentarse en reuniones médicas, pero no se revelará su identidad. Su nombre y otros datos identificativos se mantendrán confidenciales.

Esta investigación está cubierta por un Certificado de confidencialidad de los National Institutes of Health (Institutos Nacionales de Salud). Esto significa que los investigadores no pueden divulgar o usar la información, los documentos o las muestras que puedan identificarlo/a en ninguna acción legal o demanda a menos que usted lo autorice. Tampoco pueden usarlos como pruebas a menos que usted lo autorice. Esta protección incluye los procesos federales, estatales, locales, civiles, penales, administrativos, legislativos u otros. Un ejemplo sería un citatorio.

Hay algunas cosas importantes que debe saber. El Certificado NO impide la presentación de los informes que exigen las leyes federales, estatales o locales. Algunos ejemplos son leyes que requieren la denuncia de abuso infantil o abuso a mayores, el informe de ciertas enfermedades contagiosas y las amenazas de hacerse daño o hacerle daño a otros. El certificado NO PUEDE USARSE para impedir que una agencia patrocinadora gubernamental federal o estatal de los Estados Unidos revise registros o evalúe programas. El certificado NO impide las divulgaciones exigidas por la Food and Drug Administration (Administración de Alimentos y Medicamentos, o FDA, por sus siglas en inglés). Asimismo, el certificado NO impide que su información se use para otra investigación si las regulaciones federales así lo permiten.

Los investigadores pueden divulgar información sobre usted si usted lo autoriza. Por ejemplo, usted puede otorgar su autorización para divulgar su información a las aseguradoras, a los proveedores de servicios médicos o a otras personas que no estén relacionadas con la investigación. El Certificado de confidencialidad no impide que usted divulgue, voluntariamente, información sobre su participación en esta investigación. Tampoco impide que usted tenga acceso a su propia información.

El patrocinador, los monitores, los auditores, la IRB y la Food and Drug Administration tendrán acceso directo a su expediente médico para conducir y supervisar la investigación. Al firmar este documento, usted autoriza este acceso.

Las leyes federales ofrecen protecciones adicionales para sus expedientes médicos e información de salud relacionada. Estas se describen a continuación.

¿Se utilizarán mis datos o muestras para futuras investigaciones?

Como parte de este estudio, se obtendrán su información personal o muestras. Estos datos y/o muestras podrán ser utilizados por investigadores de MD Anderson y de los National Institutes of Health, o compartidos con otros investigadores y/o instituciones para su uso en

futuras investigaciones.

En algunos casos, es posible que no se elimine toda su información de identificación antes de que sus datos o muestras de investigación se usen en investigaciones futuras. Si la investigación futura se realiza en MD Anderson, los investigadores deberán obtener la aprobación de la IRB de MD Anderson antes de que se puedan usar sus datos o muestras de investigación. En ese momento, la IRB decidirá si es necesario o no obtener un permiso adicional suyo. Si la investigación en cuestión no se realiza en MD Anderson, MD Anderson no tendrá poder de supervisión sobre ningún dato ni muestra.

Si se eliminan los identificadores de su información privada o de las muestras que se obtengan en esta investigación, esa información o esas muestras podrían usarse en estudios de investigación futuros o compartirse con otros investigadores para estudios futuros de investigación sin su consentimiento informado adicional.

¿Me pueden retirar del estudio de investigación sin mi permiso?

La persona a cargo del estudio de investigación y el patrocinador pueden retirarlo/a del estudio sin su autorización. Entre los posibles motivos para la retirada se incluyen si la enfermedad empeora, si se producen efectos secundarios intolerables, si no puede seguir las instrucciones del estudio o si se interrumpe el estudio.

¿Qué ocurre si sufro una lesión por participar en este estudio?

Si se enferma o se lesiona y eso está relacionado con su participación en este estudio, le proporcionarán atención en MD Anderson (siempre que usted esté en la clínica cuando se enferme o lesione). Si se enferma o lesiona, pero no se encuentra en la clínica (por ejemplo, está en casa o en otro consultorio médico), haga lo siguiente:

- Llame a su médico/a personal de inmediato (en caso de emergencia, llame al 911).
- Informe a su médico/a personal o al personal de la sala de emergencias de que participa en este estudio (intente darles una copia de este formulario de consentimiento o muéstreles su tarjeta de participante).
- Llame al médico del estudio (Dr. David Hui, al 832-421-4450) o al 713-792-2121 (24 horas)

Ni el MD Anderson ni los National Institutes of Health le reembolsarán los gastos ni lo/la compensarán económicamente por esa lesión. Los costos del tratamiento que reciba por la enfermedad o lesión sufrida se le facturarán a usted o su compañía de seguros. No hay ninguna otra forma de pago disponible.

También puede comunicarse con la IRB de MD Anderson llamando al 713-792-6477 para preguntas sobre lesiones relacionadas con el estudio. Al firmar este formulario de consentimiento, usted no renuncia a ninguno de sus derechos legales.

¿Qué más necesito saber?

Esta investigación está financiada por los National Institutes of Health

MD Anderson podría beneficiarse de su participación o de lo que se aprenda en este estudio.

Su información (con y sin identificación) se puede usar para crear productos o prestar servicios, incluyendo algunos que pueden venderse o generar ganancias para otros. Si esto sucede, no hay planes para comunicárselo, pagarle, ni darle ninguna compensación a usted ni a su familia.

Autorización para el uso y la divulgación de la información de salud protegida (PHI, por sus siglas en inglés):

A. Durante el transcurso de este estudio, MD Anderson recopilará y usará su información de salud protegida (PHI, por sus siglas en inglés), incluida la información que puede revelar su identidad, información de su expediente médico y los resultados del estudio. Por motivos legales, éticos, de investigación y relacionados con la seguridad, su médico y el equipo de investigación pueden compartir su PHI con:
   - Las agencias federales que requieran la presentación de los datos de los estudios clínicos (como la FDA, el National Cancer Institute [Instituto Nacional del Cáncer, o NCI, por sus siglas en inglés] y la OHRP)
   - La IRB y los funcionarios de MD Anderson
   - Los National Institutes of Health, que es patrocinador o auspiciante de este estudio, o cualquier futuro patrocinador o auspiciante del estudio, y/o licenciatarios de la tecnología del estudio
   - Los monitores y los auditores del estudio que verifiquen la fidelidad de la información
   - Las personas que reúnan toda la información del estudio en informes

   Los patrocinadores o auspiciantes el estudio reciben cantidades limitadas de PHI. También podrán ver partes adicionales de PHI en los expedientes del estudio durante el proceso de monitoreo. Los contratos de MD Anderson requieren que los patrocinadores/auspiciantes protejan esta información, y limitan la forma en que la pueden utilizar.

B. Firmar este formulario de consentimiento y autorización es opcional, pero usted no podrá participar en este estudio ni recibir el tratamiento relacionado con el estudio si no está de acuerdo y no lo firma.

C. MD Anderson hará todo lo posible para proteger la privacidad de sus expedientes, pero es posible que, una vez que la información se comparta con las personas que figuran en este formulario, se divulgue a terceros. Si esto ocurre, es posible que su información deje de estar protegida por la ley federal.

D. El permiso para usar su PHI continuará vigente por tiempo indefinido, a menos que usted retire su autorización por escrito. Encontrará las instrucciones sobre cómo hacerlo en el Aviso de Prácticas de Privacidad (NPP, por sus siglas en inglés) de MD Anderson. También puede comunicarse con el/la director/a de privacidad llamando al 713-745-6636. Si retira su autorización, lo/la retirarán del estudio y los datos recopilados sobre usted hasta ese momento podrán utilizarse e incluirse en el análisis de datos. Sin embargo, no se obtendrá información adicional sobre usted.

E. En http://www.ClinicalTrials.gov encontrará una descripción de este ensayo clínico, tal como lo exigen las leyes de los Estados Unidos. Este sitio web no incluirá información que pueda identificarla/o y, a lo sumo, incluirá un resumen de los resultados. Usted podrá realizar búsquedas en este sitio web en cualquier momento.

CONSENTIMIENTO/AUTORIZACIÓN

Entiendo la información de este formulario de consentimiento. He tenido la oportunidad de leer el formulario de consentimiento de este estudio o de que alguien me lo leyera. Asimismo, he tenido la oportunidad de pensar al respecto, hacer preguntas y hablar con otras personas en la medida de lo necesario. Doy permiso al investigador principal para que me inscriba en este estudio. Al firmar este formulario de consentimiento no renuncio a ninguno de mis derechos legales. Me darán una copia firmada de este documento de consentimiento.

____ _________________
FIRMA DEL/DE LA PARTICIPANTE FECHA

____
NOMBRE DEL/DE LA PARTICIPANTE,
EN LETRA DE IMPRENTA

TESTIGO DEL CONSENTIMIENTO

Estuve presente durante la explicación de la investigación que se realizará bajo este protocolo.

____ _________________
FIRMA DEL/DE LA TESTIGO DEL CONSENTIMIENTO VERBAL (QUE NO SEA EL/LA MÉDICO/A NI EL INVESTIGADOR PRINCIPAL) FECHA

La firma de un/a testigo solo es necesaria si el/la paciente no habla inglés y utiliza el formulario de consentimiento abreviado (VTPS) o si el/la paciente no sabe leer ni escribir.

____
NOMBRE DEL/DE LA TESTIGO DEL CONSENTIMIENTO VERBAL, EN LETRA DE IMPRENTA

PERSONA QUE OBTIENE EL CONSENTIMIENTO

He hablado sobre este estudio de investigación con el/la participante y/o su representante autorizado/a, utilizando un lenguaje comprensible y apropiado. Considero que he informado completamente al/a la participante acerca de la naturaleza de este estudio, así como de sus posibles beneficios y riesgos, y que el/la participante comprendió esta explicación.

____ _________________
PERSONA QUE OBTIENE EL CONSENTIMIENTO FECHA

____
NOMBRE DE LA PERSONA QUE OBTIENE EL CONSENTIMIENTO, EN LETRA DE IMPRENTA

TRADUCTOR/A

He traducido el consentimiento informado anterior tal y como está escrito (sin añadir ni omitir información) al____________________________y he asistido a quienes
(Idioma)
obtenían y otorgaban el consentimiento traduciendo todas las preguntas y respuestas durante el procedimiento de consentimiento de este/a participante.

______________________________	___________________	_________________
NOMBRE DEL/DE LA TRADUCTOR/A	FIRMA DEL/DE LA TRADUCTOR/A	FECHA

☐ Indique si el/la traductor/a era un miembro del equipo de investigación.
(De ser así, un/a testigo, que no sea el/la traductor/a, debe firmar en la línea de testigo).

Consentimiento informado/autorización para participar en una investigación

**Título del estudio de investigación:**	Enfoque estructurado personalizado de oxígeno y terapias de apoyo para la disnea en oncología (SPOT-ON) para el tratamiento de la disnea en pacientes con cáncer: ensayo clínico aleatorizado
**Subtítulo:**	SPOT-ON
**Número del estudio:**	2023-0933
**Investigador principal:**	David Hui, MD

____________________________________ __________________________
Nombre del/de la participante | Número de expediente médico

Información clave

Este es un breve resumen del estudio para ayudarlo/a a decidir si desea participar. Más adelante en este formulario se presenta información más detallada.

¿Por qué me invitan a participar en un estudio de investigación?

Lo/la invitan a participar en un estudio de investigación porque padece un cáncer avanzado y experimenta dificultad para respirar (denominada "disnea").

¿Qué debo saber sobre un estudio de investigación?

- Una persona le explicará este estudio de investigación.
- Participar o no es su decisión.
- Puede elegir no participar.
- También puede elegir participar y cambiar de opinión más adelante.
- Su decisión no se usará en su contra.
- Puede hacer todas las preguntas que desee antes de tomar su decisión.

¿Por qué se hace esta investigación?

Actualmente sigue habiendo mucho debate sobre cuál de los tratamientos para la falta de aire es más eficaz para cada paciente. En este estudio, los pacientes probarán múltiples terapias con el fin de identificar la mejor combinación para aliviar su falta de aire.

El objetivo de este estudio de investigación clínica es conocer el efecto del tratamiento con oxígeno estructurado personalizado y las terapias de apoyo para la disnea en oncología (SPOT-ON por sus siglas en inglés) en la gravedad de la falta de aire en pacientes con cáncer.

Este es un estudio de investigación. SPOT-ON es un enfoque en investigación para decidir el mejor tratamiento de la falta de aire en pacientes con cáncer. La terapia del estudio se administrará utilizando dispositivos estándar aprobados por la FDA.

El médico del estudio podrá responder a sus preguntas sobre el tratamiento SPOT-ON.

¿Cuánto durará la investigación y qué tendré que hacer?

Recibirá el tratamiento SPOT-ON durante 72 horas (3 días). Unos 30 días después de finalizar el tratamiento, el personal del estudio comprobará cómo se encuentra.

Le pedirán que colabore con el equipo de investigación para completar el tratamiento y responder a los cuestionarios.

Encontrará información más detallada sobre los procedimientos del estudio en la sección ***“¿Qué ocurre si acepto participar en esta investigación?”***.

¿Participar en este estudio puede perjudicarme de alguna manera?

Antes de decidir participar en este estudio, hable con el equipo del estudio sobre las dudas que tenga, así como sobre los efectos secundarios, los posibles gastos y el compromiso de tiempo.

Encontrará información más detallada sobre los riesgos del estudio en el apartado ***“¿Participar en este estudio puede perjudicarme de alguna manera? (Riesgos detallados)”***.

¿Participar en este estudio me ayudará de alguna manera?

Participar en este estudio puede ayudar a aliviar su falta de aire. Los futuros pacientes podrían beneficiarse de lo que se aprenda. Sin embargo, no se puede prometer que su participación en esta investigación suponga un beneficio para usted ni para otras personas.

¿Qué ocurre si no quiero participar en esta investigación?

La participación en una investigación es totalmente voluntaria. Puede elegir participar, no participar o dejar de participar en cualquier momento sin recibir sanciones ni perder ninguno de sus beneficios habituales.

En lugar de participar en este estudio, puede optar por recibir atención estándar para la falta de aire fuera de este estudio. Puede optar por recibir otros cuidados en investigación, si están disponibles. Estas alternativas tienen riesgos y beneficios que pueden ser iguales o diferentes a los de este estudio de investigación. El médico del estudio puede hablar sobre estas alternativas con usted, incluidos sus posibles riesgos y beneficios.

En todos los casos recibirá la atención médica apropiada.

Información detallada

A continuación, encontrará información más detallada sobre este estudio, además de la información indicada anteriormente.

¿Con quién puedo hablar si tengo preguntas o inquietudes?

Si tiene preguntas, preocupaciones o quejas, o cree que la investigación lo/la ha perjudicado, hable con el equipo de investigación llamando al 713-563-7637.

Esta investigación ha sido revisada y aprobada por la Institutional Review Board (Junta de Revisión Institucional, o IRB, por sus siglas en inglés; un comité de ética que revisa los estudios de investigación) de MD Anderson. Puede hablar con ellos llamando al 713-792-6477 o escribiendo a IRB_Help@mdanderson.org si ocurre lo siguiente:

- El equipo de investigación no responde a sus preguntas, inquietudes o quejas.
- No puede comunicarse con el equipo de investigación.
- Quiere hablar con alguien que no sea parte del equipo de investigación.
- Tiene preguntas sobre sus derechos como participante en la investigación.
- Desea información o dar su opinión sobre esta investigación.

¿Cuántas personas participarán en este estudio?

Se espera que alrededor de 150 personas en MD Anderson se inscriban en este estudio de investigación.

¿Qué ocurre si acepto participar en esta investigación?

Tratamiento del estudio

Durante el tratamiento SPOT-ON de tres días, trabajará con el equipo de atención de la salud para encontrar la mejor manera de reducir su falta de aire encontrando la combinación

de tratamientos que funciona para usted. Esto incluirá probar diferentes terapias basadas en el oxígeno, como la cánula nasal, el oxígeno suplementario y la ventilación no invasiva con un terapeuta respiratorio. Además del tratamiento SPOT-ON, nuestro personal de investigación le dará cierta educación básica sobre la falta de aire y su equipo asistencial habitual le dará tratamientos estándar para su falta de aire. *Todos los pacientes del estudio comenzarán el tratamiento SPOT-ON para la falta de aire en un plazo de tres días después de su inscripción;* sin embargo, una computadora asignará aleatoriamente el momento exacto en que se iniciará este tratamiento (como al lanzar una moneda al aire).

Cuestionarios del estudio

Antes de iniciar el tratamiento, el personal del estudio recopilará información sobre sus datos demográficos (como su sexo, etnia y raza), su diagnóstico de cáncer, otras enfermedades o afecciones que pueda padecer, mediciones relacionadas con su respiración (como el nivel de oxígeno en sangre y la cantidad de aire que puede inhalar), los medicamentos que está recibiendo, otros síntomas que pueda tener y su objetivo de cuidados respiratorios. Responderá a cuestionarios sobre la intensidad y lo desagradable que le resulta su falta de aire, los síntomas que experimenta y su calidad de vida. Rellenarlos le tomará menos de 10 minutos.

Los coordinadores de la investigación evaluarán su dificultad para respirar y otros síntomas mediante cuestionarios que se realizarán por teléfono. A las 24, 48 y 72 horas, responderá los mismos cuestionarios que contestó antes de empezar este ensayo clínico, así como los cuestionarios sobre los cambios en su capacidad para respirar y su experiencia en el estudio. Rellenarlos le tomará menos de 10 minutos. El equipo del estudio también le preguntará si ha experimentado algún efecto secundario.

Seguimiento
Unos 30 días después de finalizar el tratamiento, el personal del estudio lo/la llamará para comprobar cómo se encuentra y preguntarle si ha experimentado algún efecto secundario. La llamada durará unos 5 minutos.

Si deja de participar en este estudio debido a efectos secundarios intolerables, le harán un seguimiento hasta que los efectos secundarios mejoren o se estabilicen.

¿Cuáles son mis responsabilidades si participo en esta investigación?

Si decide participar en esta investigación, sus responsabilidades serán las siguientes:

- Informar al equipo del estudio sobre cualquier síntoma o efecto secundario que tenga.
- Seguir las instrucciones del estudio.

¿Qué pasa si digo que sí, pero luego cambio de opinión?

Puede abandonar la investigación en cualquier momento, esto no se usará en su contra. Puede retirarse de la participación en este estudio sin ninguna penalización ni pérdida de

beneficios. Aunque se retire de este estudio, podrá elegir recibir tratamiento en MD Anderson.

Si decide que quiere dejar de participar en el estudio, se recomienda por su seguridad que hable primero con su médico, quien puede ayudarlo/a a interrumpir el tratamiento del estudio de forma segura. Puede ser peligroso interrumpir repentinamente el tratamiento del estudio. El médico del estudio también decidirá si necesita someterse a alguna visita o prueba para controlar su salud.

Si deja de participar en el estudio de investigación, los datos ya recogidos no podrán eliminarse de la base de datos del estudio. Es posible que le pregunten si el médico del estudio puede recopilar datos de su atención médica de rutina. Si está de acuerdo, estos datos se manejarán igual que los de la investigación.

¿Participar en este estudio puede perjudicarme de alguna manera? (Riesgos detallados)

Mientras participe en este estudio, usted corre el riesgo de tener efectos secundarios, Estos efectos secundarios variarán de una persona a otra. Los efectos secundarios más comunes se encuentran enumerados en este formulario, al igual que los poco frecuentes, pero graves. Debe hablar sobre ellos con el médico del estudio. También le sugerimos que pregunte acerca de los efectos secundarios poco comunes que se hayan observado en un número escaso de pacientes, pero que no estén enumerados en este formulario. Muchos efectos secundarios desaparecen poco después del procedimiento, pero en algunos casos pueden ser graves, duraderos o permanentes, y pueden incluso requerir hospitalización y/o causar la muerte.

Informe al personal del estudio de cualquier efecto secundario que pueda tener, incluso si considera que no está relacionado con el procedimiento.

Efectos secundarios del uso de oxígeno suplementario (cánula nasal de alto flujo, ventilación no invasiva y oxígeno suplementario de bajo flujo)

La cánula nasal de alto flujo, la ventilación no invasiva y el oxígeno suplementario de bajo flujo consisten en suministrarle oxígeno adicional. El uso de oxígeno suplementario puede provocar lo siguiente:

• irritación o sequedad en la boca o la garganta<br>• irritación de los ojos (posible sequedad de los ojos o enrojecimiento doloroso de los ojos)	• dificultad para respirar<br>• irritación o sequedad en la nariz	• toxicidad del oxígeno (posibles daños en los pulmones, los ojos y el sistema nervioso, sobre todo con una exposición prolongada a altos niveles de oxígeno)

Existe un mayor riesgo de incendio, especialmente si el/la paciente fuma o si el oxígeno se escapa del dispositivo de suministro y entra en contacto con una fuente de ignición.

Efectos secundarios de la cánula nasal de alto flujo

La cánula nasal de alto flujo puede provocar sangrado nasal y un mayor riesgo de infección, como la neumonía. Esta infección puede ocurrir en cualquier parte y poner en peligro la vida. Los síntomas de infección pueden incluir fiebre, dolor, enrojecimiento y dificultad para respirar.

Efectos secundarios de la ventilación no invasiva

La ventilación no invasiva puede provocar los siguientes efectos secundarios:

• molestias por la mascarilla o irritación de la piel detrás de las orejas o debajo de la nariz<br>• dificultad para tolerar el soporte de presión, como hambre de aire, dolor torácico o hinchazón abdominal	• aumento del trabajo respiratorio o de la resistencia de las vías respiratorias, que puede provocar el fracaso de la ventilación no invasiva y la necesidad de ventilación invasiva	• empeoramiento de la afección respiratoria o cardiaca subyacente, como una mayor necesidad de oxígeno o la presencia de líquido en el pulmón<br>• infección

La ventilación no invasiva puede provocar un mayor riesgo de infección, como neumonía o sinusitis. Esta infección puede ocurrir en cualquier parte y poner en peligro la vida. Los síntomas de infección pueden incluir fiebre, dolor, enrojecimiento y dificultad para respirar.

Otros riesgos

Los **cuestionarios** pueden contener preguntas de naturaleza sensible. Usted puede negarse a responder cualquier pregunta que le haga sentir incomodidad. Si tiene alguna pregunta acerca de cómo contestar el cuestionario, le aconsejamos que contacte a su médico o al investigador principal.

Aunque se tomarán todas las medidas posibles para mantener la seguridad de los datos del estudio, existe la posibilidad de que su información de salud personal se pierda o sea robada, lo que puede dar lugar a una **pérdida de confidencialidad**. Todos los datos del estudio se almacenarán en computadoras protegidas por contraseña o armarios cerrados con llave, y permanecerán almacenados de manera segura después del estudio.

Además de estos riesgos, esta investigación puede perjudicarlo/a de formas que aún se desconocen. Esto podría ser desde una inconveniencia leve hasta una consecuencia tan grave que ocasione la muerte.

Le comunicaremos cualquier información nueva que pudiera afectar su salud, su bienestar o su deseo de permanecer en este estudio.

¿Cuál será el costo de participar en este estudio?
¿Me pagarán por participar en este estudio?

Los tratamientos SPOT-ON ofrecidos por el/la terapeuta respiratorio le serán proporcionados de forma gratuita para usted.

Ni usted ni su compañía de seguros tendrán que pagar por ciertos procedimientos y exámenes de investigación que estén cubiertos por el estudio.

Usted y/o su compañía de seguros serán responsables de los costos de los servicios clínicos de rutina (como procedimientos diagnósticos/terapéuticos, medicamentos, dispositivos, ensayos de laboratorio y otros servicios que normalmente se solicitarían para la atención médica, independientemente de si participa o no en un estudio). Puede haber otros costos que no cubra su plan médico y que deberá pagar usted.

Participar en este estudio puede suponerle costos añadidos (como transporte, estacionamiento, comidas o permisos no remunerados en el trabajo). Es posible que tenga que pagar la medicación que le receten para tratar o prevenir los efectos secundarios y que tenga que acudir a la clínica o al hospital con más frecuencia que si no participara en este estudio.

Si tiene seguro, hable con su compañía de seguros y asegúrese de que entiende lo que paga su seguro y lo que no paga si participa en este estudio. Además, averigüe si necesita la aprobación previa de su plan para poder participar en el estudio.

Usted puede pedir que le pongan a su disposición un/a asesor/a de finanzas para que hable con usted sobre los costos de este estudio.

Como compensación por su tiempo y esfuerzo, recibirá una tarjeta regalo de 50 dólares por completar el estudio.

¿Qué ocurre con la información recopilada para la investigación?

Se hará todo lo posible para limitar el uso y la divulgación de su información personal, incluidos los expedientes médicos y del estudio de investigación, a las personas que necesitan revisar esta información. No podemos garantizar el secreto total. Entre las organizaciones que pueden inspeccionar y copiar su información, se incluyen la IRB y otros representantes de esta organización.

Una vez se haya inscrito en el estudio, le asignarán un número de participante del estudio. Este número de participante del estudio se utilizará para identificar sus datos en el informe del estudio y al comunicar cualquier dato del estudio.

Se eliminará o modificará cualquier información personal que permita su identificación antes de que se compartan los datos con otros investigadores o de que se hagan públicos los resultados.

Los resultados de esta investigación podrán publicarse en revistas científicas o presentarse en reuniones médicas, pero no se revelará su identidad. Su nombre y otros datos identificativos se mantendrán confidenciales.

Esta investigación está cubierta por un Certificado de confidencialidad de los National Institutes of Health (Institutos Nacionales de Salud). Esto significa que los investigadores no pueden divulgar o usar la información, los documentos o las muestras que puedan identificarlo/a en ninguna acción legal o demanda a menos que usted lo autorice. Tampoco pueden usarlos como pruebas a menos que usted lo autorice. Esta protección incluye los procesos federales, estatales, locales, civiles, penales, administrativos, legislativos u otros. Un ejemplo sería un citatorio.

Hay algunas cosas importantes que debe saber. El Certificado NO impide la presentación de los informes que exigen las leyes federales, estatales o locales. Algunos ejemplos son leyes que requieren la denuncia de abuso infantil o abuso a mayores, el informe de ciertas enfermedades contagiosas y las amenazas de hacerse daño o hacerle daño a otros. El certificado NO PUEDE USARSE para impedir que una agencia patrocinadora gubernamental federal o estatal de los Estados Unidos revise registros o evalúe programas. El certificado NO impide las divulgaciones exigidas por la Food and Drug Administration (Administración de Alimentos y Medicamentos, o FDA, por sus siglas en inglés). Asimismo, el certificado NO impide que su información se use para otra investigación si las regulaciones federales así lo permiten.

Los investigadores pueden divulgar información sobre usted si usted lo autoriza. Por ejemplo, usted puede otorgar su autorización para divulgar su información a las aseguradoras, a los proveedores de servicios médicos o a otras personas que no estén relacionadas con la investigación. El Certificado de confidencialidad no impide que usted divulgue, voluntariamente, información sobre su participación en esta investigación. Tampoco impide que usted tenga acceso a su propia información.

El patrocinador, los monitores, los auditores, la IRB y la Food and Drug Administration tendrán acceso directo a su expediente médico para conducir y supervisar la investigación. Al firmar este documento, usted autoriza este acceso.

Las leyes federales ofrecen protecciones adicionales para sus expedientes médicos e información de salud relacionada. Estas se describen a continuación.

¿Se utilizarán mis datos o muestras para futuras investigaciones?

Como parte de este estudio, se obtendrán su información personal o muestras. Estos datos y/o muestras podrán ser utilizados por investigadores de MD Anderson y de los National Institutes of Health, o compartidos con otros investigadores y/o instituciones para su uso en

futuras investigaciones.

En algunos casos, es posible que no se elimine toda su información de identificación antes de que sus datos o muestras de investigación se usen en investigaciones futuras. Si la investigación futura se realiza en MD Anderson, los investigadores deberán obtener la aprobación de la IRB de MD Anderson antes de que se puedan usar sus datos o muestras de investigación. En ese momento, la IRB decidirá si es necesario o no obtener un permiso adicional suyo. Si la investigación en cuestión no se realiza en MD Anderson, MD Anderson no tendrá poder de supervisión sobre ningún dato ni muestra.

Si se eliminan los identificadores de su información privada o de las muestras que se obtengan en esta investigación, esa información o esas muestras podrían usarse en estudios de investigación futuros o compartirse con otros investigadores para estudios futuros de investigación sin su consentimiento informado adicional.

¿Me pueden retirar del estudio de investigación sin mi permiso?

La persona a cargo del estudio de investigación y el patrocinador pueden retirarlo/a del estudio sin su autorización. Entre los posibles motivos para la retirada se incluyen si la enfermedad empeora, si se producen efectos secundarios intolerables, si no puede seguir las instrucciones del estudio o si se interrumpe el estudio.

¿Qué ocurre si sufro una lesión por participar en este estudio?

Si se enferma o se lesiona y eso está relacionado con su participación en este estudio, le proporcionarán atención en MD Anderson (siempre que usted esté en la clínica cuando se enferme o lesione). Si se enferma o lesiona, pero no se encuentra en la clínica (por ejemplo, está en casa o en otro consultorio médico), haga lo siguiente:

- Llame a su médico/a personal de inmediato (en caso de emergencia, llame al 911).
- Informe a su médico/a personal o al personal de la sala de emergencias de que participa en este estudio (intente darles una copia de este formulario de consentimiento o muéstreles su tarjeta de participante).
- Llame al médico del estudio (Dr. David Hui, al 832-421-4450) o al 713-792-2121 (24 horas)

Ni el MD Anderson ni los National Institutes of Health le reembolsarán los gastos ni lo/la compensarán económicamente por esa lesión. Los costos del tratamiento que reciba por la enfermedad o lesión sufrida se le facturarán a usted o su compañía de seguros. No hay ninguna otra forma de pago disponible.

También puede comunicarse con la IRB de MD Anderson llamando al 713-792-6477 para preguntas sobre lesiones relacionadas con el estudio. Al firmar este formulario de consentimiento, usted no renuncia a ninguno de sus derechos legales.

¿Qué más necesito saber?

Esta investigación está financiada por los National Institutes of Health

MD Anderson podría beneficiarse de su participación o de lo que se aprenda en este estudio.

Su información (con y sin identificación) se puede usar para crear productos o prestar servicios, incluyendo algunos que pueden venderse o generar ganancias para otros. Si esto sucede, no hay planes para comunicárselo, pagarle, ni darle ninguna compensación a usted ni a su familia.

Autorización para el uso y la divulgación de la información de salud protegida (PHI, por sus siglas en inglés):

A. Durante el transcurso de este estudio, MD Anderson recopilará y usará su información de salud protegida (PHI, por sus siglas en inglés), incluida la información que puede revelar su identidad, información de su expediente médico y los resultados del estudio. Por motivos legales, éticos, de investigación y relacionados con la seguridad, su médico y el equipo de investigación pueden compartir su PHI con:
   - Las agencias federales que requieran la presentación de los datos de los estudios clínicos (como la FDA, el National Cancer Institute [Instituto Nacional del Cáncer, o NCI, por sus siglas en inglés] y la OHRP)
   - La IRB y los funcionarios de MD Anderson
   - Los National Institutes of Health, que es patrocinador o auspiciante de este estudio, o cualquier futuro patrocinador o auspiciante del estudio, y/o licenciatarios de la tecnología del estudio
   - Los monitores y los auditores del estudio que verifiquen la fidelidad de la información
   - Las personas que reúnan toda la información del estudio en informes

   Los patrocinadores o auspiciantes el estudio reciben cantidades limitadas de PHI. También podrán ver partes adicionales de PHI en los expedientes del estudio durante el proceso de monitoreo. Los contratos de MD Anderson requieren que los patrocinadores/auspiciantes protejan esta información, y limitan la forma en que la pueden utilizar.

B. Firmar este formulario de consentimiento y autorización es opcional, pero usted no podrá participar en este estudio ni recibir el tratamiento relacionado con el estudio si no está de acuerdo y no lo firma.

C. MD Anderson hará todo lo posible para proteger la privacidad de sus expedientes, pero es posible que, una vez que la información se comparta con las personas que figuran en este formulario, se divulgue a terceros. Si esto ocurre, es posible que su información deje de estar protegida por la ley federal.

D. El permiso para usar su PHI continuará vigente por tiempo indefinido, a menos que usted retire su autorización por escrito. Encontrará las instrucciones sobre cómo hacerlo en el Aviso de Prácticas de Privacidad (NPP, por sus siglas en inglés) de MD Anderson. También puede comunicarse con el/la director/a de privacidad llamando al 713-745-6636. Si retira su autorización, lo/la retirarán del estudio y los datos recopilados sobre usted hasta ese momento podrán utilizarse e incluirse en el análisis de datos. Sin embargo, no se obtendrá información adicional sobre usted.

E. En http://www.ClinicalTrials.gov encontrará una descripción de este ensayo clínico, tal como lo exigen las leyes de los Estados Unidos. Este sitio web no incluirá información que pueda identificarla/o y, a lo sumo, incluirá un resumen de los resultados. Usted podrá realizar búsquedas en este sitio web en cualquier momento.

CONSENTIMIENTO/AUTORIZACIÓN

Entiendo la información de este formulario de consentimiento. He tenido la oportunidad de leer el formulario de consentimiento de este estudio o de que alguien me lo leyera. Asimismo, he tenido la oportunidad de pensar al respecto, hacer preguntas y hablar con otras personas en la medida de lo necesario. Doy permiso al investigador principal para que me inscriba en este estudio. Al firmar este formulario de consentimiento no renuncio a ninguno de mis derechos legales. Me darán una copia firmada de este documento de consentimiento.

FIRMA DEL/DE LA PARTICIPANTE FECHA

NOMBRE DEL/DE LA PARTICIPANTE,
EN LETRA DE IMPRENTA

TESTIGO DEL CONSENTIMIENTO

Estuve presente durante la explicación de la investigación que se realizará bajo este protocolo.

FIRMA DEL/DE LA TESTIGO DEL CONSENTIMIENTO VERBAL (QUE NO SEA EL/LA MÉDICO/A NI EL INVESTIGADOR PRINCIPAL) FECHA

La firma de un/a testigo solo es necesaria si el/la paciente no habla inglés y utiliza el formulario de consentimiento abreviado (VTPS) o si el/la paciente no sabe leer ni escribir.

NOMBRE DEL/DE LA TESTIGO DEL CONSENTIMIENTO
VERBAL, EN LETRA DE IMPRENTA

PERSONA QUE OBTIENE EL CONSENTIMIENTO

He hablado sobre este estudio de investigación con el/la participante y/o su representante autorizado/a, utilizando un lenguaje comprensible y apropiado. Considero que he informado completamente al/a la participante acerca de la naturaleza de este estudio, así como de sus posibles beneficios y riesgos, y que el/la participante comprendió esta explicación.

PERSONA QUE OBTIENE EL CONSENTIMIENTO FECHA

NOMBRE DE LA PERSONA QUE OBTIENE EL
CONSENTIMIENTO, EN LETRA DE IMPRENTA

<u>TRADUCTOR/A</u>

He traducido el consentimiento informado anterior tal y como está escrito (sin añadir ni omitir información) al____________________________y he asistido a quienes
(Idioma)
obtenían y otorgaban el consentimiento traduciendo todas las preguntas y respuestas durante el procedimiento de consentimiento de este/a participante.

____________________________	____________________	__________________
NOMBRE DEL/DE LA TRADUCTOR/A	FIRMA DEL/DE LA TRADUCTOR/A	FECHA

☐ Indique si el/la traductor/a era un miembro del equipo de investigación.
(De ser así, un/a testigo, que no sea el/la traductor/a, debe firmar en la línea de testigo).
