# Supplementary material for: Structured Personalized Oxygen and Supportive Therapies for Dyspnea in Oncology (SPOT-ON): A personalized randomized clinical trial protocol
Source: PLoS One. 2025 Dec 2;20(12):e0336691. doi: 10.1371/journal.pone.0336691 (PMC12671826; doi:10.1371/journal.pone.0336691)
Supplement: S4 File — (PDF) [file pone.0336691.s004.pdf]

Data Safety Monitoring Board (DSMB)

The DSMB is an officially constituted committee of the University of Texas MD Anderson (MD Anderson) that is designed to oversee the data safety monitoring of clinical trials. The primary objectives of the DSMB are to: 1) ensure that participants in a trial are protected; 2) ensure that participants' interests are not made secondary to the interests of the scientific investigation; and 3) monitor all clinical trials that originate at MD Anderson or that are coordinated or analyzed by MD Anderson.

The DSMB has the following responsibilities: 1) to review interim analyses of outcome data (prepared by the study statistician or another responsible person at the time points defined in the protocol approved by the institutional review board [IRB]) and to recommend, if necessary, that the study be changed or terminated; 2) to determine whether and to whom outcome results should be released prior to the study results being reported; 3) to review interim toxicity and efficacy data; 4) to review major modifications to the study proposed by the principal investigator (PI) prior to implementation (e.g., termination, dropping an arm because of toxicity, or increasing the target sample size); and 5) to communicate with the appropriate persons at MD Anderson about issues that may arise and provide effective resolutions for participant care and risk prevention as well as educational purposes.

The DSMB is composed of scientists and statisticians from within and/or outside the institution, selected on the basis of their experience, reputation for objectivity, absence of conflicts of interest, and knowledge of good clinical trial methods. The DSMB members represent participant interests, not those of the institution.

The DSMB meets at least once a year, and more often if necessary. The PI may prepare a report that addresses specific toxicity concerns or other concerns about the conduct of the study during the open session. A copy of the statistician's report may be sent to the DSMB chair for presentation during the closed portion but not to any other individuals not on the DSMB. The report may contain recommendations on whether to close the study, whether to report the results, whether to continue accrual or follow-up, and whether DSMB discussion is needed.

The review of each trial includes three parts. The first part is an open session at which members of the study team may be present, at the request of the DSMB, to answer questions. In this part, the focus is on accrual, compliance, and toxicity issues. After this open session, the second session is a closed session in which the DSMB discusses the interim outcome results by treatment arm with the study statisticians. Finally, the third session, the executive session, is limited to DSMB members, alternates, and ex officio members.

DSMB recommendations are based on results for the current study being monitored and on data available to the DSMB from other related studies. The PI will ensure that the DSMB is advised about relevant non-confidential results from other related studies that become available. It will be the responsibility of the DSMB to determine the extent to which this information is relevant to decisions to continue or modify the current study. The DSMB will provide recommendations in writing to the PI to change or stop a study or a part thereof (e.g., one arm) or to continue the study unchanged, with special consideration given to participants already in treatment. Copies of such communication will be preserved in the official committee minutes.

In the event that a study change is recommended for participant safety reasons (including early stopping of inferior therapy), the PI implements the change as expeditiously as possible to ensure participants' safety. In the unlikely situation that the PI does not concur with the DSMB recommendation, the Vice President for Research Administration is informed of the DSMB's recommendation and the PI's reason for disagreeing with the recommendation. The Vice President for Research Administration, the DSMB Chair, Office of Human Subjects Protection staff, and the PI will conduct the appeal process. The Vice President for Research Administration will make the final decision regarding the appeal request. Confidentiality is maintained during these discussions. In the event that a change in a study is recommended for reasons other than participant safety (e.g., to extend accrual because of a lower than expected accrual rate), the PI is responsible for preparing and submitting an amendment to the IRB that includes the DSMB's recommendations and the rationale for the changes. IRB approval of the amendment will be required prior to implementing the change, although a decision to override the DSMB's recommendation is made only in the most exceptional circumstances.

All documents, investigative reports, information, and conversations relating to the committee's work are strictly confidential and are not shared with anyone other than committee members. Although committee documents are subject to legal privileges as set forth in statutory and case law and are not subject to discovery during a litigation process, the privilege may be lost if committee documents are given to, shown to, or discussed with non-committee members without an official DSMB request to do so.

No communication about the DSMB's deliberations or recommendations, written or oral, occurs outside of the committee, except as provided for in these policies and procedures. All DSMB members or alternates sign statements of confidentiality at the beginning of an appointment period. Outcome (efficacy) results are strictly

confidential and are not divulged to non-members (excepting the PI and Associate Vice President for Clinical Investigations) until the recommendation to report the results are accepted and implemented.

Individuals invited to serve on the DSMB disclose to the group chair any potential (real or perceived) conflicts of interest. These include professional interest, proprietary interest, and miscellaneous interest considerations. Potential conflicts that develop during the conduct of a trial should also be disclosed to the PI.

Open Session: During the open session of the DSMB, voting and non-voting members of the DSMB as well as the clinical trial team may be present, at the request of the DSMB Chair. The PI and other appropriate study leadership as well as the protocol biostatistician(s) should be in attendance in order to present results and respond to questions. The focus of the open session is on the general conduct and progress of the study. Specifically, the focus of the open session includes adverse events and toxicity issues, subject accrual, protocol compliance, demographic characteristics of enrollees, disease status of enrollees (if relevant), site performance, quality control, and timeliness and completeness of follow-up. During this time, no confidential data will be discussed and the blind, if present, will be maintained.

Closed Session: The second part of the meeting is a closed session involving the voting members as well as any ex officio members invited at the discretion of the DSMB Chair. External involvement in the closed session is against MD Anderson DSMB bylaws. During this part of the meeting, grouped safety data and, if appropriate, efficacy data to include unmasking of blinded data are presented by the protocol biostatistician(s).

Executive Session: The third part of the meeting involves only voting DSMB members and provides an opportunity to discuss the general conduct of the trial, all outcome results, including toxicities and adverse events, and implications of data. External involvement in the executive session is against MD Anderson DSMB bylaws. The Chair, if applicable, may break the blind, if such action is required to make an informed decision. Recommendations will be made to continue the study as planned, to make adjustments to the study plan, or to suspend or terminate the study. At the end of the meeting, voting members discuss and vote on these recommendations. Voting may be conducted by voice/show of hands or by ballot. Every effort will be made to obtain a consensus. If consensus cannot be obtained, a majority vote is required to carry any recommendation. The Chair will participate in discussions.
